# Does type or diversity of activities delay aging-related cognitive decline?

**DOI:** 10.1093/geroni/igaf133

**Published:** 2026-02-05

**Authors:** Dana A Glei, Chioun Lee, Casey K Brown, Maxine Weinstein

**Affiliations:** Center for Population and Health, Georgetown University, Washington, District of Columbia, United States; Department of Sociology, University of California, Riverside, Riverside, California, United States; Department of Psychology, Georgetown University, Washington, District of Columbia, United States; Center for Population and Health, Georgetown University, Washington, District of Columbia, United States

**Keywords:** United States, Cognition, Health and Retirement Study, Midlife in the United States Study

## Abstract

**Background and Objectives:**

Research has shown a correlation between engagement in activities and late-life cognition, but cross-sectional associations are likely to be inflated by reverse causality. This study investigated the prospective effects of activity engagement—frequency of and diversity across activity types—on aging-related cognitive decline.

**Research Design and Methods:**

Using data from the Health and Retirement Study (HRS) and Midlife in the United States (MIDUS) study, we evaluated whether baseline measures of 4 activity types (cognitive, physical, contact with family/friends, and social group participation) predicted subsequent cognitive decline adjusted for potential confounders. We compared the effects of activity type frequency with the effect of activity diversity.

**Results:**

In HRS, activity diversity was associated with slower midlife (ages 55-65) cognitive decline, whereas more frequent cognitive activities yielded the largest reduction in late-life (ages 65-85) cognitive decline. Frequency of social contact was associated with slower midlife cognitive decline, whereas more frequent social group participation had a stronger association in later life. Physical activity did not significantly affect the cognitive decline trajectory. In MIDUS, neither the activity frequency nor diversity was associated with subsequent cognitive decline.

**Discussion and Implications:**

Our results underscore that both type and timing of activity matter: Efforts to promote activity diversity and social contact are likely to be most effective in midlife, whereas cognitive activities and social group participation may be more impactful in late life. Physical activity alone had little effect on mid-to-late-life cognition but may be valuable earlier in life and in the context of activity diversity.

Innovation and Translational Significance: Cognitive, social, or physical activities may benefit cognition, but individuals with better cognition can be more willing or able to engage in such activities. We used prospective analysis with rigorous adjustment for confounders to address potential reverse causality that can bias observational studies. Both the type and timing of activity matter: activity diversity and social contact may delay midlife cognitive decline, whereas cognitive activities and social group participation may be more impactful in late life. The positive effect of doing many different activities on the rate of cognitive decline during midlife was nearly as large as the negative effect of smoking.

## Background and objectives

Cognitive impairment and dementia are growing public health concerns, particularly given population aging. Recent estimates suggest that 42% of Americans may develop dementia in their lifetime.^1^ Thus, there is increasing interest in modifiable risk factors that might reduce or delay cognitive decline even in midlife.

The Lancet Commission on dementia highlights cognitive, physical, and social activity among 14 modifiable protective factors for dementia.^2^ Unlike other risk factors that are genetic, generally determined early in life (eg, education), or require costly interventions (eg, hearing aids, treatments for diabetes, or hypertension), engagement in activity is potentially modifiable even in mid- and late-life and can be adapted across diverse settings and populations for relatively low cost.

The potential benefits of engagement in activities on cognitive health are supported by 2 theoretical frameworks that explain how such behaviors may enhance or preserve brain function. Cognitive Reserve Theory suggests that cognitive, physical, and social activity can make cognitive processes more resilient by influencing the capacity, efficiency, or flexibility of brain networks.^3-5^ Similarly, the Scaffolding Theory of Aging and Cognition (STAC) posits that such activities can enhance brain function (eg, efficient connectivity, synaptic density) and increase compensatory scaffolding (ie, supplemental neural circuitry that helps counteract neurofunctional decline in an aging brain).^6^

There are also indirect mechanisms through which activity engagement affects neurodegeneration. For example, physical activity benefits cardiovascular health,^7^ which can reduce the risk of vascular dementia. Insufficient physical and social activity may also heighten the stress response (ie, activation of the hypothalamic-pituitary-adrenal axis and the sympathetic nervous system) and systemic inflammation, which can lead to neuroinflammation (ie, chronic immune response of the brain).^7^^,^^8^ In addition, social interaction may influence other behaviors (eg, smoking, alcohol consumption) that contribute to vascular disease, though the effect could be positive (eg, better health behaviors) or negative (eg, greater alcohol consumption in the context of social activity).^8^

A large literature has demonstrated cross-sectional associations between various types of activities (cognitive, physical, and social) and late-life cognition,^9-11^ but those relationships are likely to be inflated by reverse causality (ie, cognition may influence a person’s willingness and ability to engage in particular activities). That caveat is especially notable for cognitively demanding activities but may also be salient for social activities, which require the ability to read social cues and interact appropriately, and for physical activity, which relies on the ability to self-regulate.

If the relationship between activities and late-life cognition is not causal but merely a result of confounding or reverse causality, then interventions that focus on activity engagement would be ineffective in preserving cognitive function. Instead, public policy efforts should prioritize interventions that have greater potential. Randomized experiments are the gold standard for establishing causality, but they are often constrained by limited resources, ethical considerations, and problems of generalizability. In older populations, there are additional logistical and methodological challenges when conducting randomized trials with long-term follow-up. Consequently, most research on this topic relies on observational studies. When using survey data to evaluate the extent to which activities may have a causal effect on cognitive decline in late life, it is crucial to conduct prospective analyses with rigorous adjustment for potential confounders.

Many prior prospective studies investigate the link between various types of activities and subsequent cognitive decline, but there is considerable variation across studies in the range of activities included. For example, when measuring social activity, some researchers combine a broad range of activities defined as potentially social. These activities may include in-person visits with friends and/or family,^12-14^ social group participation,^12^^,^^14-16^ religious attendance,^12-14^ volunteer work,^12-14^ entertainment,^12-14^ and, in some cases, day/overnight trips,^13^^,^^14^ working for pay after full retirement,^12^ or informal caregiving.^12^ Hsieh et al.^17^ focus on group activity but separately model the effects of social, networking, religious, and volunteer participation on cognitive decline. Others focus more specifically on social contact with family and friends.^18^^,^^19^

In addition to social activity, some studies explicitly include cognitively stimulating activities,^13^^,^^14^^,^^19^ while a few also include physical activity.^16^^,^^18^^,^^20^ Other studies focus exclusively on cognitive activities^21^ or physical activity.^15^^,^^22-26^ One study combines a large set of disparate measures into a single enrichment score—including cognitive, physical, and social activities, but also educational attainment, bilingualism, knowledge of a foreign language, household size, whether the respondent’s job is enriching, and hobbies—making it impossible to determine whether the effect is driven by a particular type of activity or by one of the other factors such as educational attainment.^27^

Other research suggests that diversity across activity types may particularly benefit cognition by requiring individuals to shift between different tasks and adapt to new situations, which activates the hippocampus and may help maintain brain plasticity and increase cognitive reserve.^28-30^ Among the studies that measured activity diversity, most were cross-sectional.^28^^,^^30-35^ We found only one prospective study: Carlson et al.^29^ reported that the variety of activities has a stronger effect on the incidence of cognitive impairment than the overall frequency of activities. However, because their frequency measure aggregated all activities rather than distinguishing specific types, it is difficult to determine how the observed benefits of diversity compare with the effects of frequent engagement in particular types of activities. Few studies have explicitly compared the effects of activity frequency by domain (cognitive, physical, and social) versus activity diversity on aging-related cognitive decline.

If activities affect the rate of cognitive decline with age, it implies that their effect on the level of cognition differs by age. Prior work has demonstrated that cognitive decline accelerates at the oldest ages, and protective factors do not necessarily slow cognitive decline indefinitely,^36^ which implies that the effect of activities on the rate of cognitive decline might also differ by age. As Aarsten et al.^36^ demonstrated for childhood socioeconomic conditions, a group may have early-life cognitive advantages, but, at some point, cognitive reserve can no longer compensate for aging-related neuronal loss. Eventually, changes in the brain that typically accompany aging and the accumulation of underlying pathology may accelerate decline even for those who were cognitively advantaged earlier in life. Consequently, they begin to catch up with the less advantaged group. It is reasonable to expect a similar pattern for activity engagement. Higher frequency of activities or more diverse activities may slow cognitive decline at younger ages, but that benefit could diminish or even reverse at advanced ages as neurodegeneration overwhelms cognitive reserve.^37^ More specific evidence about the type of activities most likely to preserve cognition at which stages of life could help inform effective, targeted interventions.

Using data from 2 national surveys with overlapping age ranges—the Midlife in the United States study (MIDUS, ages 33-84) and the Health and Retirement Study (HRS, ages 50+)—we evaluate whether type or diversity of activities might have a causal effect on aging-related cognitive decline. We aim to appropriately model the age function of cognition and leverage the full depth of the available data to address the problems of confounding and reverse causality that have plagued many prior observational studies. Specifically, we investigate the relationship prospectively using *baseline* measures of various types of activities to predict *subsequent* cognitive decline adjusted for a broad set of potential confounders. Given the variation across previous studies in how social activities are classified, we measure social contact and group participation separately so that we can explicitly compare their effects. Thus, we categorize activities into 4 domains: cognitive, physical, engagement with social contacts, and social group participation. Then, we compare the effects of the frequency of different types of activities with the effect of diversity across activity types.

## Research design and methods

### Data

We used data from 2 U.S. national longitudinal surveys: HRS and MIDUS. Supplementary Table 1 (see online supplementary material) summarizes the sample designs, response rates, and restrictions on the analytic sample for each survey. One advantage of MIDUS is that the sampling frame targets a younger population, who are less likely to have already experienced substantial cognitive decline. Using multiple datasets also helps us evaluate the robustness of results across independent samples and ensures that the findings are not merely the result of a peculiarity in a particular sample.^38^

#### HRS

HRS began in 1992 with follow-up waves every 2 years and refresher cohorts added every 6 years; see Supplementary Methods Section 1 (see online supplementary material) for more details. This analysis used data from Waves 2008-2020 because 2008 was the first wave that included the cognitive activity questions.

We limited our analysis to the age-eligible sample (ie, cohorts aged 50 or older at their initial interview). The cohorts targeted in the sampling design were aged 54 and older in 2008 (because they were originally sampled in 2004 or earlier), aged 50 years and older in 2010 and 2016 (when younger cohorts were sampled), and aged 54 years and older by 2020.

The cognitive and social activity data came from the psychosocial and lifestyle self-administered questionnaire (SAQ), which is separate from the main interview. Therefore, we excluded respondents who never completed that SAQ. We also excluded institutionalized respondents from HRS to enhance comparability with MIDUS. Individuals interviewed by proxy were also excluded from this analysis because they were not administered cognitive tests.

Our analytic sample comprised 20,817 respondents who were interviewed up to 7 times over the 2008-2020 waves, totaling 86,567 observations. The number of respondents ranged from 6,479 (2008) to 14,488 (2012), with an average of 3.0 waves per person.

#### MIDUS

The MIDUS study began in 1995-96 (Wave 1); see Supplementary Methods Section 2 (see online supplementary material) for more details. Approximately 9 years later, at Wave 2, a new sample in Milwaukee was added to the study. At each wave, the initial interview (by phone or computer-assisted personal interviewing) was followed by a mail-in self-administered questionnaire (SAQ). Starting at Wave 2, a cognitive battery was administered in a separate phone interview. We restricted analyses to the 2713 respondents who completed the SAQ at Wave 2 and cognitive testing at Waves 2 (aged 33-84 years at cognitive testing in 2004-2006) and 3 (aged 42-94 in 2013-2018). The median interval between cognitive testing at Waves 2 and 3 was 9.6 years.

### Measures

#### Cognitive function

The outcome measure was overall cognition, based on the Telephone Interview for Cognitive Status (TICS) in HRS^39^ and the Brief Test of Adult Cognition by Telephone (BTACT) in MIDUS.^40^^,^^41^ The cognitive tests evaluated episodic memory (ie, immediate and delayed recall) and executive function (eg, backward counting, subtraction, verbal fluency; see Supplementary Methods Section 3, see online supplementary material, for more details). In both surveys, higher scores indicate better cognition.

#### Frequency of activities

We constructed measures of the frequency of 4 types of activities: (1) cognitive; (2) physical; (3) contact within social network; and (4) social group participation.

##### Cognitive

In both HRS and MIDUS, there were 6 questions regarding cognitive activities (ie, reading books/magazines/newspapers, doing word games, such as crossword puzzles or scrabble, playing cards or other games such as bridge/chess, attending educational lectures/courses, writing, using a computer such as to send e-mail or search the Internet), each coded on a 6-point scale from never to daily. We recoded each item to the approximate frequency per month (never = 0, less than once a week = 2, once a week = 4, several times a week = 8, daily = 30) and then summed across all 6 items to derive a measure of the overall frequency of cognitive activities per month (range = 0-180).

##### Physical

Both HRS and MIDUS asked about the frequency of physical activity at different levels of intensity (light/mild, moderate, vigorous). However, one difference between the 2 studies is that MIDUS asked separate questions for summer and winter and for different contexts (work, home chores, leisure). Also, MIDUS used 6 response categories (maximum = “several times per week”), whereas HRS used only 5 response categories (maximum = “daily”). We converted the measures from their original ordinal scale to the approximate frequency per month (MIDUS: never/hardly ever = 0, less than once a month = 0.5, once a month = 1, once a week = 4, several times a week = 8; HRS: hardly ever/never = 0, 1-3 times per month = 2, once a week = 4, more than once a week = 8, daily = 30).

For MIDUS, we computed the average across summer and winter for each level of intensity (vigorous, moderate, and light activity) by context (work, home chores, and leisure). Then, we summed the values across the 3 contexts to obtain a measure of the overall frequency of physical activity at each level of intensity (to maximize comparability with HRS). Finally, in both datasets, we summed across all intensities to derive the overall frequency of physical activity (range = 0-90 in HRS; 0-72 in MIDUS).

##### Contact within social network

HRS and MIDUS differed somewhat in the format of the questions about social contact with various members of the respondent’s social network (see Supplementary Methods Section 4, see online supplementary material, for more details). For both HRS and MIDUS, we used all relevant items for contact with family members (living outside the household) or friends and converted each item to the approximate frequency of contact per month. Then, we summed across all items to obtain the overall frequency (per month) of contact within the respondent’s social network (range = 0-108 in HRS; 0-120 in MIDUS).

##### Social group participation

Questions about the frequency of social group participation (eg, sport/social/other clubs, meetings with other groups, religious/spiritual service attendance) also differed somewhat between HRS and MIDUS (see Supplementary Methods Section 5, see online supplementary material, for more details). Within each dataset, we summed across items to obtain the overall frequency of social group participation per month. Final scores ranged from 0-68 in HRS and 0-79 in MIDUS.

#### Diversity of activities

We used an adaption of Shannon’s diversity index^42^ to measure the diversity of activities:


$$H_{i}=-\sum_{j}^{J}\left[(p_{ij})\times \ln\left(p_{ij}\right)\right],$$

where $p_{ij}$ denotes the frequency (per month) for respondent i of activity type j (cognitive, physical, social contacts, and social group participation) as a proportion of the overall frequency of activities. Then, we divided $H_{i}\;$ by the maximum of $H_{i}$ [ie, the logarithm of the number of activity types—in this case, ln(4)] so that the resulting index ranged from 0 to 1 (where 1 = equal distribution of the monthly frequencies across the 4 activity types).

Because of variation in measurement, the maximum frequency differed by type of activity and across surveys. To ensure that each type of activity was given equal weight when computing diversity, we top-coded the frequency per month to 60 or more (ie, twice daily). The percentage of respondents who were top-coded for the purposes of computing diversity was highest for cognitive activities (47% in MIDUS; 39% in HRS), followed by social contacts per month (30% in MIDUS; 9% in HRS). The percentages were lower for physical activity (5% in both MIDUS and HRS) and social group participation (<0.1% in MIDUS; <0.2% in HRS).

#### Confounders

We controlled for potential confounders expected to affect both activity levels and cognition. Sociodemographic confounders comprised age, sex, race/ethnicity, education, marital status (married vs all else), and employment status. We also included established risk factors for dementia^2^: history of a head injury, smoking history (never, former, current), alcohol consumption, and various measures of health status (ie, hearing, vision, diabetes, hypertension, high cholesterol, obesity, and depression). We controlled for the baseline levels of these variables as well as the history of heart problems, the history of stroke, physical limitations, and personality traits. See Supplementary Methods Section 6, see online supplementary material, for details about the measurement of confounders. A directed acyclic graph depicting the hypothesized relationships for the prospective analyses is shown in Supplementary Figure 1 (see online supplementary material).

### Analytic strategy

All analyses were conducted in Stata 18.5. We used multiple imputation to handle missing data; see Supplementary Methods Section 7, see online supplementary material, for details.

Descriptive statistics for the baseline covariates are presented in Supplementary Table 2 (see online supplementary material). Baseline was defined as the first available observation for HRS (2008 for cohorts born before 1954; 2010 for those born in 1954-1959; 2016 for those born in 1960-1965) and 2004-2006 for MIDUS.

For HRS, we used a random slope (ie, growth curve) model to estimate the effects of activities on the age trajectory of late-life cognition (ie, predictors were allowed to affect both the level of cognition at baseline and the rate of cognitive decline with aging). We used age as the time metric for the growth curve model because age is the most important determinant of mid- to late-life cognition and because study participants were observed for different segments of the age range (eg, some cohorts were observed only from age 50 to 56, whereas others were observed only from 76 to 90). The Huber–White estimator was used to correct the standard errors for clustering by primary sampling unit.

For MIDUS, we could not fit a growth curve model because we have only 2 waves of cognitive testing. Instead, we first estimated a cross-sectional model that regressed cognition on activities at Wave 2. Second, we estimated a prospective model testing the association between activities measured at Wave 2 and subsequent changes in cognition (Wave 3—Wave 2). Given the wide variation in the length of the interval between Waves 2 and 3 cognitive testing (mean = 9.6 years, range = 7.5-13.6), we adjusted the observed change in cognition to represent the implied change after 10 years of aging (eg, divided by the length of the interval between the 2 cognitive tests and multiplied by 10). Because MIDUS sampled multiple respondents per family, we used the Huber–White estimator to correct the standard errors for intra-family correlation.

When there are only 2 longitudinal measures of the outcome, some prior studies have used a lagged dependent variable (LDV) model. However, Glymour^43^ demonstrated that a change score model is the most appropriate method for evaluating the causal effect of a key predictor on subsequent change in the dependent variable when the baseline measurement (LDV) is a potential mediator, as is the case here (ie, earlier levels of activities may have already affected baseline cognition). See Supplementary Methods Section 8 (see online supplementary material) for more details regarding the choice of modeling strategy.

The results from the cross-sectional MIDUS model can be compared with the HRS growth curve model estimates for the association between baseline measures of activities and the level of cognition (ie, intercept), whereas the estimates from the prospective MIDUS model can be compared with the HRS growth curve model estimates for the effect of baseline activities on the rate of subsequent cognitive decline (ie, slope). The prospective results are more likely to support a causal interpretation.

For both datasets, Model 1 included measures of the frequency of 4 types of activities (cognitive, physical, social contacts, and social groups) adjusted only for age. We used a quadratic specification for age because prior work has demonstrated that cognitive decline accelerates at the oldest ages.^36^ Correlations among the frequencies of the 4 types of activities were weak (*r *≤ 0.21). Even the 2 measures of social activity (social contacts and group participation) were weakly correlated with each other (*r *= 0.16 in HRS, 0.13 in MIDUS). Model 2 further adjusted for a broad range of other potential confounders: sociodemographic characteristics, health behaviors (ie, smoking history, alcohol consumption), health status (ie, history of a head injury, hearing, vision, hypertension, taking medication for high cholesterol, obesity, selected chronic health conditions, physical limitations, depression), and personality traits. We did not include activity diversity in the same model with the frequencies of different types of activities because diversity was strongly correlated with social group participation (*r *= 0.47 in HRS, 0.57 in MIDUS). Thus, it would be difficult to estimate their independent effects (eg, high social group participation in the absence of high diversity). Instead, we substituted activity diversity in place of the frequencies of different activities in Model 3.

In HRS, interactions between the activity variables and age reflect the effects of those variables on the rate of aging-related cognitive decline. If activities are associated with the rate of cognitive decline, it implies that the effect of activities on the *level* of cognition varies by age. If the age trajectory were linear, then the effect of a predictor on the *slope* of cognitive decline would be the same regardless of age. However, when fitting a quadratic age trajectory, the effect of a predictor on the rate of cognitive decline can also vary by age because interactions with both the linear and quadratic terms for age mean the predictor can affect the shape of the age trajectory.

In MIDUS, we tested age interactions with the activity variables (for comparability with the growth curve model for HRS). None of the interactions between the activity variables and age (linear and quadratic terms) were jointly significant. Therefore, we omitted those interactions from the final models.

## Results

### Cross-sectional associations

Cross-sectionally, cognitive and physical activities were positively associated with cognition in the age-adjusted models (Tables 1 and 2, Model 1). The measures of social activity (social contact and social group participation) were weakly associated with cognition after controlling for other activities. Even the bivariate correlations with baseline cognition were very weak for social contact (*r *= 0.05 in HRS, −0.01 in MIDUS) and social group participation (*r* = 0.02 in HRS, 0.01 in MIDUS) but stronger for cognitive activities (*r *= 0.30 in HRS, 0.28 in MIDUS) and physical activity (*r *= 0.11 in HRS, 0.20 in MIDUS). In auxiliary models that tested social contact and social group participation individually controlling only for age (not shown), the cross-sectional associations with cognition were in the expected positive direction, although social contact was not significant in MIDUS. After further adjustment for other types of activities, most of the associations became negative, albeit still weak.

**Table 1.** Coefficients (95% CIs) from growth curve models predicting age trajectory of cognition, Health and Retirement Study 2008-2020 (*N *= 86,567 observations).

Variable	(1)	(2)	(3)
**Age (decades after 65)^a^**	−0.157***	−0.160***	−0.174***
	(−0.173, −0.140)	(−0.203, −0.117)	(−0.218, −0.130)
**Age (decades after 65) squared**	−0.137***	−0.104***	−0.104***
	(−0.144, −0.129)	(−0.131, −0.077)	(−0.131, −0.077)
**Effects on cognition at age 65**			
**Cognitive activity^b^ ^,c^**	0.307***	0.096***	
	(0.293, 0.322)	(0.084, 0.109)	
**Physical activity^b^ ^,c^**	0.079***	0.010	
	(0.063, 0.096)	(−0.003, 0.022)	
**Social contact^b^ ^,c^**	−0.011	0.010	
	(−0.023, 0.002)	(−0.003, 0.023)	
**Social group participation^b^ ^,c^**	−0.028**	−0.016*	
	(−0.046, −0.010)	(−0.030, −0.003)	
**Activity diversity^c^**			0.064***
			(0.053, 0.076)
**Effects on rate of cognitive decline**			
**Cognitive activity^c^ × age**	0.016*	0.018**	
	(0.003, 0.029)	(0.007, 0.030)	
**Cognitive activity^c^ × age^2^**	−0.002	0.004	
	(−0.010, 0.005)	(−0.004, 0.011)	
**Physical activity^c^ × age**	0.004	0.000	
	(−0.008, 0.015)	(−0.010, 0.011)	
**Physical activity^c^ × age^2^**	−0.005	−0.001	
	(−0.013, 0.002)	(−0.009, 0.006)	
**Social contact^c^ × age**	0.020**	0.015*	
	(0.007, 0.033)	(0.003, 0.027)	
**Social contact^c^ × age^2^**	−0.009*	−0.006	
	(−0.017, −0.001)	(−0.014, 0.002)	
**Group participation^c^ × age**	0.013	0.005	
	(−0.000, 0.025)	(−0.007, 0.016)	
**Group participation^c^ × age^2^**	0.008	0.007	
	(−0.000, 0.016)	(−0.000, 0.015)	
**Activity diversity^c^ × age**			0.018**
			(0.006, 0.030)
**Activity diversity^c^ × age^2^**			−0.009**
			(−0.015, −0.003)
**Constant**	0.180***	0.232***	0.212***
	(0.156, 0.203)	(0.192, 0.271)	(0.172, 0.252)

*Note*. Model 1 is adjusted only for age. Models 2 and 3 are further adjusted for baseline measures of sociodemographic characteristics, health behaviors (ie, smoking history, alcohol consumption), health status, and personality traits, as well as the interactions between those variables and age (quadratic) to account for their effects on the rate of cognitive decline. Model 3 substitutes activity diversity in place of the frequencies of different activities. The full model results are shown in Supplementary Table 3 (see online supplementary material).

a Age is measured in decades after age 65: (age—65/10); thus, the coefficient represents the effect per 10 years of aging.

b Frequency per month.

c Standardized so that the coefficient represents the effect per *SD*.

* *p *< .05.

** *p *< .01.

*** *p *< .001.

**Table 2.** Coefficients (95% CIs) from cross-sectional regression of cognition on activities (both measured at Wave 2), Midlife in the United States Study (*N *= 2713).

Variable	(1)	(2)	(3)
**Age (decades after 65)^a^**	−0.374***	−0.348***	−0.347***
	(−0.423, −0.325)	(−0.404, −0.291)	(−0.404, −0.290)
**Age (decades after 65) squared**	−0.030*	−0.031**	−0.036**
	(−0.054, −0.006)	(−0.054, −0.009)	(−0.059, −0.014)
**Cognitive activity^b^ ^,c^**	0.285***	0.165***	
	(0.250, 0.319)	(0.132, 0.198)	
**Physical activity^b^ ^,c^**	0.090***	0.036*	
	(0.054, 0.125)	(0.003, 0.070)	
**Social contact^b^ ^,c^**	−0.037*	−0.004	
	(−0.073, −0.001)	(−0.037, 0.029)	
**Social group participation^b^ ^,c^**	0.020	0.007	
	(−0.015, 0.054)	(−0.023, 0.037)	
**Activity diversity^c^**			0.059***
			(0.026, 0.092)
**Constant**	−0.299***	−0.499***	−0.579***
	(−0.346, −0.252)	(−0.623, −0.375)	(−0.704, −0.453)

*Note*. Model 1 is adjusted only for age. Models 2 and 3 are further adjusted for baseline measures of sociodemographic characteristics, health behaviors (ie, smoking history, alcohol consumption), health status, and personality traits. Model 3 substitutes activity diversity in place of the frequencies of different activities. The full model results are shown in Supplementary Table 4 (see online supplementary material).

a Age is measured in decades after age 65: (age—65/10); thus, the coefficient represents the effect per 10 years of aging.

b Frequency per month.

c Standardized so that the coefficient represents the effect per *SD*.

* *p *< .05.

** *p *< .01.

*** *p *< .001.

However, a substantial share of the age-adjusted correlations appeared to result from confounding (eg, with educational attainment). After adjustment for other potential confounders (Model 2), the associations with cognitive activity were substantially attenuated. The association with physical activity was virtually eliminated in HRS and diminished by more than half in MIDUS. The negative association with social group participation in HRS was further weakened, while the negative association with social contact in MIDUS disappeared.

In Model 3, when we substituted activity diversity in place of the frequencies of different activity types, it was also positively associated with cognition in both datasets (HRS: standardized $\hat{\beta }$ = 0.06 at age 65, 95% CI: 0.05**-**0.08; MIDUS: $\hat{\beta }$ = 0.06, 95% CI: 0.03**-**0.09), but the magnitude was weaker than that of the frequency of cognitive activities in Model 2 (HRS: $\hat{\beta }$ = 0.10, 95% CI: 0.08**-**0.11; MIDUS: $\hat{\beta }$ = 0.17, 95% CI: 0.13**-**0.20).

### Prospective effects

**Figure 1.**
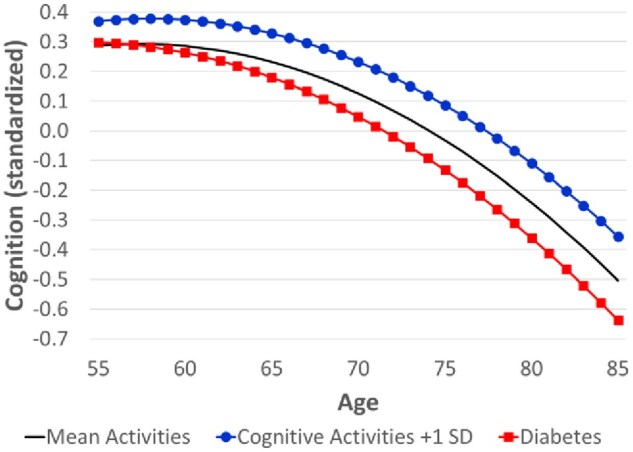
Estimated age trajectory of cognition by selected levels of cognitive activities and diabetes, Health and Retirement Study. *Note*. The y-axis represents cognition (standardized to represent *SD* units). The estimates are based on the coefficients in Model 2 (Table 1). The black line represents the reference group (ie, mean levels of all continuous variables—which includes activity frequencies; the omitted category for all other binary and categorical variables). For the line marked by blue circles, all values remain unchanged except for the specified difference in cognitive activities. The line marked by red squares denotes diabetes (while those without diabetes are represented by the reference group).

The prospective results indicate the effect of activities on subsequent cognitive decline, and thus, provide stronger evidence that the effect may be causal. Based on the fully-adjusted model for HRS, more frequent cognitive activities were associated with a slower rate of cognitive decline (as demonstrated by the interaction with age; Table 1, Model 2). Given the difficulty of interpreting interactions with both linear and quadratic age terms in the HRS model, Table 3 shows the estimated cognitive decline between selected ages for different levels of activity. Compared with the reference group (ie, average frequency for all activity domains), those who engaged in more frequent cognitive activities (at baseline) had slower (subsequent) cognitive decline, particularly above age 65. The estimated age trajectories in Figure 1 show that more frequent cognitive activities were associated with better cognition (cross-sectionally) and slower cognitive decline (prospectively). Consequently, the gap widened with age (from 0.08 *SD* at age 55 to 0.15 *SD* at age 85).

**Table 3.** Estimated levels of cognition and cognitive decline between selected ages by specified level of various activities, Health and Retirement Study.

Variable	Cognition at age 55	Cognitive decline across 10-year age intervals:			Cognition at age 85
		55-65	65-75	75-85	
**Based on Model 2:**					
** Mean frequencies of activities^a^ *[reference group]***	0.287	−0.055	−0.264	−0.472	−0.504
** Frequency by activity type**					
** Cognitive activities: 1 *SD* above mean^b^**	0.369***	−0.041	−0.242***	−0.443**	−0.357***
** Physical activity: 1 *SD* above mean^b^**	0.295	−0.054	−0.265	−0.004	−0.500
** Social contacts: 1 *SD* above mean^b^**	0.276	−0.035*	−0.255	−0.003	−0.489
** Social group participation: 1 *SD* above mean^b^**	0.273	−0.058	−**0.252** *	−0.447**	−0.484
** Diabetes**	0.298	−0.119**	−0.312***	−0.505	−0.638***
**Based on Model 3:**					
** Mean activity diversity^a^ *[reference group]***	0.283	−0.071	−0.278	−0.485	−0.551
** Activity diversity: 1 *SD* above mean^b^**	0.320***	−0.043**	−0.269	−0.495	−0.487***
** Current smoker**	0.243	−0.101	−0.313	−0.525	−0.696**

*Note*. Estimated values of cognition are expressed in *SD* units. The estimates for the frequency of different types of activities are based on Model 2, whereas the estimates for diversity are based on Model 3 (Table 1). Values that were significantly better (higher level of cognition or slower cognitive decline) than the reference group are shown in bold. Values that were significantly worse (lower level of cognition or faster cognitive decline) than the reference group are shown in italics.

a Represents the reference group with average levels of all continuous variables (including the frequency of all activity domains or diversity) and the omitted category for binary and categorical variables (ie, non-Hispanic White men who were married, completed high school or GED, currently employed, never smoked, never drink alcohol, do not have hypertension, do not take medication for high cholesterol, and did not have heart problems, a history of a stroke, or diabetes).

b Same as above except for the specified difference in the indicated activity measure.

* *p *< .05.

** *p *< .01.

*** *p *< .001 relative to the reference group.

In Figure 1, we also show the trajectory for diabetics (relative to non-diabetics, represented by the reference group) as a benchmark for comparing effect sizes. There was little difference between diabetics and non-diabetics in the level of cognition at age 55, but diabetes was associated with faster cognitive decline. As a result, the gap in cognition associated with diabetes widened from 0.01 *SD* at age 55 to −0.13 *SD* at age 85.

Physical activity had no significant effect on the rate of cognitive decline (Table 3). As further demonstrated in Supplementary Figure 2 (see online supplementary material), the overall effect of physical activity on the age trajectory of cognition was negligible.

As for social activity, more frequent social contact was associated with slower cognitive decline at younger ages (eg, 55-65), while higher frequency of social group participation was associated with slower decline at older ages (eg, 65-85; Table 3). However, Supplementary Figure 3 (see online supplementary material) suggests that the magnitude of those effects was small.

**Figure 2.**
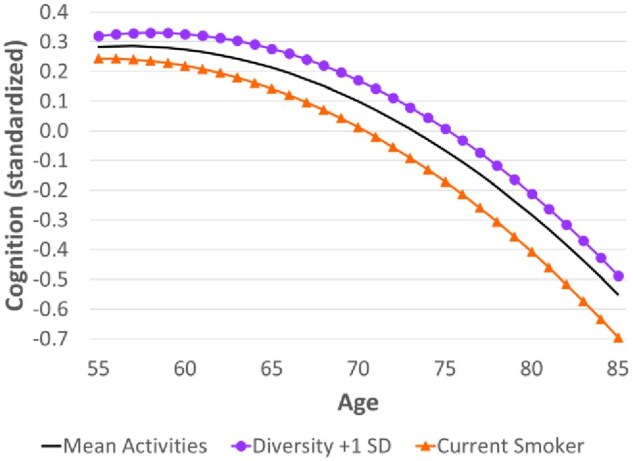
Estimated age trajectory of cognition by selected levels of activity diversity and smoking, Health and Retirement Study. *Note*. The y-axis represents cognition (standardized to represent *SD* units). The estimates are based on the coefficients in Model 3 (Table 1). The black line represents the reference group (ie, mean levels of all continuous variables—which includes activity diversity; the omitted category for all other binary and categorical variables). For the line marked by purple circles, all values remain unchanged except for the specified difference in activity diversity. The line marked by orange triangles denotes current smokers (while never smokers are represented by the reference group).

Activity diversity was associated with slower cognitive decline at younger ages (eg, 55-65; Table 3) but that benefit diminished with age. Figure 2 shows that the difference in cognition by level of diversity widened between age 55 (0.04 *SD*) and 75 (0.07 *SD*) but narrowed slightly after that (to 0.06 *SD* at age 85). Even at age 85, those with more diverse activities still exhibited better cognition (−0.49 *SD* below the mean) than the reference group (−0.55 *SD* below the mean).

In Figure 2, we also present the trajectory for current smokers (relative to never smokers as the reference group) to compare the magnitude of the effect size with that of diversity. At younger ages, the association between activity diversity and the rate of cognitive decline was similar to the difference between never and current smokers, although the effect of smoking did not diminish with age. Thus, the gap between current and never smokers widened from −0.04 *SD* at age 55 to −0.14 *SD* at age 85.

In MIDUS (Supplementary Table 5, see online supplementary material), none of the activity variables—neither frequencies by activity type (Model 2) nor activity diversity (Model 3)—was significantly associated with subsequent cognitive decline. Supplementary Table 6 (see online supplementary material) shows estimated levels of cognition at ages 35 and 85 (based on cross-sectional models in Table 2) and the estimated cognitive decline over 10 years starting at ages 35, 45, 55, 65, and 75 (based on the prospective models in Supplementary Table 5, see online supplementary material) for different levels of activity. There were significant cross-sectional differences in the level of cognition but no significant prospective effects on subsequent cognitive decline.

## Discussion and implications

This study investigated the prospective effects of activity engagement—including the frequency of different types of activities as well as diversity across activity types—on aging-related cognitive decline. We focus on the prospective results because we cannot infer the direction of causality from cross-sectional associations: although activity engagement may benefit cognition, it is equally possible that individuals with better cognition were more willing or able to engage in activities. If so, the cross-sectional association would be inflated by reverse causality.

Although we did not have any explicit life-course hypotheses, our prospective models based on HRS suggested that activity diversity was associated with slower cognitive decline at younger ages, but that effect diminished with age. Despite slight convergence in trajectories at the oldest ages, individuals with more diverse activities still maintained better levels of cognition even at age 85, consistent with previous cross-sectional studies that found activity diversity associated with better cognition.^28^^,^^30-35^ We found one prospective study based on the Women’s Health and Aging Study that included diversity, but it focused on cognitive impairment rather than the age trajectory of cognitive decline, aggregated the frequency of all activities, and did not evaluate whether the effect varied by age.^29^

The “catching up” pattern for activity diversity that we observed in HRS is consistent with cognitive reserve theory and with prior research on other protective factors, such as childhood socioeconomic conditions^36^ and educational attainment.^37^ Factors associated with slower cognitive decline at younger ages may change direction at the oldest ages because cognitive reserve can no longer compensate for aging-related ­neuronal loss.

Among those aged 65 years and older, our results from HRS implied that more frequent cognitive activity was most strongly associated with the rate of cognitive decline. Previous studies based on MIDUS^18^ and HRS^20^ reported that cognitively stimulating activities were associated with slower cognitive decline,^18^^,^^20^ while another study of Black adults in the Minority Aging Research Study (MARS) reported no significant effect.^14^ None of the prior studies allowed for accelerated decline at the oldest ages or assessed whether the effect on the rate of cognitive decline varied by age. In MIDUS, we did not find a significant prospective effect for cognitive activity, whereas Sharifian et al.^18^ did, but their results should be interpreted with caution because they used a lagged dependent variable model.

Our prospective models based on HRS suggested that social contact was associated with slower cognitive decline at younger ages, whereas social group participation had a stronger association at older ages. We are not aware of any prior studies that examined separately these 2 aspects of social activity or evaluated whether the effects on cognitive decline differed by age. Studies based on MIDUS^18^ and HRS^19^ reported that social contact with friends (but not family) was associated with slower cognitive decline, but those models did not include social group participation. We did not find a significant prospective effect of social contact in MIDUS, even in preliminary models that examined the effects of contact with friends and family separately. Again, we suspect that differences in modeling strategy may explain the discrepancy between our results and Sharifian et al.^18^ They used a lagged dependent variable model, which is likely to inflate the association. Among the studies that defined social activity more broadly, an analysis based on MARS found a significant association with slower cognitive decline,^14^ while 2 studies based on HRS did not.^12^^,^^20^

Our finding that physical activity had no significant effect on the rate of cognitive decline is consistent with Greendale et al.,^22^ who concluded that the association between physical activity and late-life cognition may be an artifact of reverse causation. Villalonga et al.^20^ also found no significant effect of physical activity on cognitive decline net of cognitive and social activities. Other studies have reported physical activity to be associated with slower cognitive decline,^15^^,^^18^^,^^24^^,^^26^^,^^44^ but that may be a result of differences in modeling strategy (eg, failure to account for accelerated cognitive decline at the oldest ages; limited adjustment for potential confounders; biased estimates because of adjustment for the lagged dependent variable).

Another possibility is that most of the benefits of physical activity were already realized earlier in life. Even if physical activity enhances cognitive development and the accumulation of cognitive reserve earlier in life, it is difficult to alter the trajectory of cognitive decline in late life. Thus, the incremental effect on *future* cognitive decline may be negligible. That does not necessarily imply that physical activity has no cognitive benefit, but it does suggest that an intervention to increase these activities in late life may have slim prospects for slowing future cognitive decline. It may be more effective to maintain activity levels throughout early adulthood and midlife, *before* there is a notable aging-related cognitive decline.

Staying physically active throughout adulthood may also help prevent chronic diseases (eg, diabetes, cardiovascular disease) and maintain physical function as individuals age, which, in turn, can affect cognition. Our models adjust for physical limitations and major chronic diseases, as these may inhibit activity levels. But physical inactivity earlier in life could have contributed to the development of diabetes and cardiovascular disease, causing a stroke that impaired physical and cognitive function in late life. In that scenario, physical inactivity might be a root cause, but increasing physical activity late in life cannot reverse damage that has already been done. In contrast, those who were physically active throughout adulthood may have been better able to preserve their cognitive function. For them, discontinuing or reducing physical activity could accelerate cognitive decline.

### Limitations

In MIDUS, the lack of significant effects from the prospective models may stem from (1) more limited follow-up (only one follow-up, approximately 9 years later vs up to 6 follow-ups every 2 years in HRS) and (2) a smaller sample size (more than 30 times as many observations in HRS as in MIDUS). Detecting differences in the rate of late-life cognitive decline requires substantial statistical power, as these differences are typically very small. Given accelerated cognitive decline with aging, at least 4 waves of longitudinal data are required to estimate within-individual quadratic age trajectories. In the future—with additional follow-ups—it would be possible to appropriately model individual-level age trajectories of cognition based on MIDUS.

A second limitation of this study is that activity data are self-reported. Individuals with worse cognition are more prone to misreporting, which may bias the results.

Third, individuals who experienced severe cognitive impairment were likely to be omitted from analysis or lost to follow-up because proxy respondents were not administered the cognitive tests and because the institutionalized population was excluded. Even with 2-year survey intervals in HRS, we could miss rapid cognitive decline if those with worse cognition were less likely to participate in the survey and more likely to die before they could be reinterviewed. Consequently, our results are more likely to capture early cognitive decline than the later stages of impairment and dementia.

Finally, despite our best efforts to address potential reverse causality with prospective analysis, the effect of cognitive activities may still be overestimated (ie, engagement in cognitively demanding activities may be a result of underlying cognitive health and confounders associated with cognition). Our results do not appear to be consistent with the meta-analysis of randomized controlled trials, which concluded that cognitive activity had no effect on the performance of various cognitive tests.^45^ If we had measures of cognition early in life—well before there was any notable aging-related cognitive decline—perhaps we could control for the effects of early-life cognition (eg, before age 25) on activity engagement in midlife. Then, we could obtain a better estimate of the net effect of midlife activity levels (eg, at age 35) on the subsequent trajectory of cognition in later life (eg, from age 35 to 85). Data from longitudinal studies that follow individuals from a young age (eg, Add Health, National Longitudinal Survey of Youth, or Dunedin Study) could eventually allow such analyses when those cohorts reach late life.

## Conclusion

Using data from 2 U.S. national surveys, MIDUS and HRS, we compare the prospective effects of different types of activities and activity diversity on cognitive decline from midlife to old age. Importantly, we attempted to address the issues of confounding and reverse causality that often bias observational studies. In MIDUS, neither the frequency nor diversity of activities was associated with subsequent cognitive decline. Our findings from HRS suggested that engaging in a diverse range of activities was associated with slower cognitive decline during midlife, whereas more frequent participation in cognitive activities was most strongly associated with slower cognitive decline in late life.

The effects of the social activity variables also differed by age: at younger ages, the frequency of social contact was associated with slower cognitive decline, whereas more frequent social group participation had a stronger association at older ages. By itself, physical activity had little effect on mid- to late-life cognition, but it may be valuable earlier in life and in the context of activity diversity.

The positive effect of engaging in many different activities on the rate of cognitive decline during midlife was nearly as large as the negative effect of smoking, although the association with activity diversity diminished with age, whereas the effect of smoking did not. At older ages, the effect size for cognitive activities was notably smaller than that of diabetes. Our results underscore that both the type and timing of activity matter: Efforts to promote activity diversity and social contact are likely to be most effective in midlife, whereas cognitive activities and social group participation may be more impactful in late life.

## Supplementary Material

igaf133_Supplementary_Data

## Data Availability

The analyses reported in this manuscript were not preregistered. The data for HRS can be accessed at https://hrs.isr.umich.edu/data-products/. We used the 2022 Cross-Wave Tracker File (Early, Version 2, November 2024), the RAND HRS Longitudinal File 2022 (version 1, May 2025), the RAND HRS Fat files for 2008-2020, and the Langa-Weir Classification of Cognition Function 1995-2020 (version 2, May 2023). The data for MIDUS can be accessed at https://midus.colectica.org/ or https://www.icpsr.umich.edu/web/ICPSR/series/203. We used the data from MIDUS 1 (Core) for Project 1 (main survey, version 19, doi:10.3886/ICPSR02760.v19); from MIDUS 2 (Core) for Project 1 (main survey, version 8, doi:10.3886/ICPSR04652.v8), Project 3 (Cognitive, version 7, doi:10.3886/ICPSR25281.v7), and the Milwaukee survey (MKE1, version 7, doi:10.3886/ICPSR22840.v7); from MIDUS 3 (Core) for Project 1 (main survey, version 7, doi:10.3886/ICPSR36346.v7), Project 3 (Cognitive, version 3, doi:10.3886/ICPSR37095.v3), and the Milwaukee survey (MKE2, version 4, doi:10.3886/ICPSR37120.v4); and the Mortality file for the Core Sample (2/4/2025 version, https://midus-study.github.io/public-documentation/Mortality/Core/MIDUS_Core_MortalityCauseData_N2533_20250204.sav).

## References

[1] Fang M , Hu J, Weiss J, et al. Lifetime risk and projected burden of dementia. Nat Med. 2025;31:772-776. 10.1038/s41591-024-03340-9 39806070 PMC12305800

[2] Livingston G , Huntley J, Liu KY, et al. Dementia prevention, intervention, and care: 2024 report of the Lancet standing Commission. Lancet. 2024;404:572-628. 10.1016/S0140-6736(24)01296-0 39096926

[3] Cabeza R , Albert M, Belleville S, et al. Maintenance, reserve and compensation: the cognitive neuroscience of healthy ageing. Nat Rev Neurosci. 2018;19:701-710. 10.1038/s41583-018-0068-2 30305711 PMC6472256

[4] Tucker AM , Stern Y. Cognitive reserve in aging. Current Alzheimer Research. 2011;8:354-360. 10.2174/156720511795745320 21222591 PMC3135666

[5] Pettigrew C , Soldan A. Defining cognitive reserve and implications for cognitive aging. Curr Neurol Neurosci Rep. 2019;19:1. 10.1007/s11910-019-0917-z 30627880 PMC7812665

[6] Reuter-Lorenz PA , Park DC. How does it STAC up? Revisiting the scaffolding theory of aging and cognition. Neuropsychol Rev. 2014;24:355-370. 10.1007/s11065-014-9270-9 25143069 PMC4150993

[7] Huuha AM , Norevik CS, Moreira JBN, et al. Can exercise training teach us how to treat Alzheimer’s disease? Ageing Research Reviews. 2022;75:101559. 10.1016/j.arr.2022.101559 34999248

[8] Sommerlad A , Kivimäki M, Larson EB, et al. Social participation and risk of developing dementia. Nat Aging. 2023;3:532-545. 10.1038/s43587-023-00387-0 37202513

[9] Evans IEM , Martyr A, Collins R, Brayne C, Clare L. Social isolation and cognitive function in later life: a systematic review and meta-analysis. J Alzheimers Dis. 2019;70(Suppl 1):S119-S144. 10.3233/JAD-180501 30372678 PMC6700717

[10] Iso-Markku P , Aaltonen S, Kujala UM, et al. Physical activity and cognitive decline among older adults: a systematic review and meta-analysis. JAMA Network Open. 2024;7:e2354285. 10.1001/jamanetworkopen.2023.54285 38300618 PMC10835510

[11] Yates LA , Ziser S, Spector A, Orrell M. Cognitive leisure activities and future risk of cognitive impairment and dementia: systematic review and meta-analysis. Int Psychogeriatr. 2016;28:1791-1806. 10.1017/S1041610216001137 27502691

[12] Cabrera-Haro L , Mendes de Leon CF. Retirement, social engagement, and post-retirement changes in cognitive function. J Aging Health. 2024:8982643241308311. 10.1177/08982643241308311 39703034

[13] James BD , Wilson RS, Barnes LL, Bennett DA. Late-life social activity and cognitive decline in old age. J Int Neuropsychol Soc. 2011;17:998-1005. 10.1017/S1355617711000531 22040898 PMC3206295

[14] Nsor NA , Bourassa KJ, Barnes LL, Brown CK. The effects of APOE alleles, cognitive activities, and social activities on cognitive decline in African Americans. The J Geron B Psychol Sci Soc Sci. 2024;80:gbae172. 10.1093/geronb/gbae172 PMC11632228 39392924

[15] Kraal AZ , Dotterer HL, Sharifian N, et al. Physical activity in early- and mid-adulthood are independently associated with longitudinal memory trajectories in later life. J Gerontol A Biol Sci Med Sci. 2021;76:1495-1503. 10.1093/gerona/glaa252 33000124 PMC8277086

[16] Stephan Y , Sutin AR, Luchetti M, Aschwanden D, Terracciano A. Physical, cognitive, and social activities as mediators between personality and cognition: evidence from four prospective samples. Aging & Mental Health. 2024;28:1294-1303. 10.1080/13607863.2024.2320135 38410951 PMC11324381

[17] Hsieh M , Yang TO, Li T, et al. Types of social group participation and long-term cognitive preservation in older ages. Innovation in Aging. 2025;9:igaf009. 10.1093/geroni/igaf009 40297772 PMC12036325

[18] Sharifian N , Kraal AZ, Zaheed AB, Sol K, Zahodne LB. Longitudinal associations between contact frequency with friends and with family, activity engagement, and cognitive functioning. J Int Neuropsychol Soc. 2020;26:815-824. 10.1017/S1355617720000259 32200766 PMC7483134

[19] Zahodne LB , Ajrouch KJ, Sharifian N, Antonucci TC. Social relations and age-related change in memory. Psychol Aging. 2019;34:751-765. 10.1037/pag0000369 31180697 PMC6710103

[20] Villalonga-Olives E , Majercak KR, Almansa J, Khambaty T. Longitudinal impact of volunteering on the cognitive functioning of older adults: a secondary analysis from the US Health and Retirement Study. Int J Nurs Sci. 2023;10(3):373-382. 10.1016/j.ijnss.2023.06.018 37545782 PMC10401338

[21] Stieger M , Lachman ME. Increases in cognitive activity reduce aging-related declines in executive functioning. Front Psychiatry. 2021;12:708974. 10.3389/fpsyt.2021.708974 34393863 PMC8358146

[22] Greendale GA , Han W, Huang M, et al. Longitudinal assessment of physical activity and cognitive outcomes among women at midlife. JAMA Network Open. 2021;4:e213227. 10.1001/jamanetworkopen.2021.3227 33787912 PMC8013795

[23] Hamer M , Muniz G, Demakakos P. Physical activity and trajectories in cognitive function: English Longitudinal Study of Ageing. J Epidemiol Community Health. 2018;72:477-483. 10.1136/jech-2017-210228 29434025 PMC5977988

[24] Hong C , Liu Z, Liu Y, Jin Y, Luo Y. The role of smoking, obesity, and physical inactivity in cognitive performance and decline: a multicohort study. J Gerontol A Biol Sci Med Sci. 2024;79:glad232. 10.1093/gerona/glad232 37778005

[25] James SN , Chiou YJ, Fatih N, Needham LP, Schott JM, Richards M. Timing of physical activity across adulthood on later-life cognition: 30 years follow-up in the 1946 British birth cohort. J Neurol Neurosurg Psychiatry. 2023;94:349-356. 10.1136/jnnp-2022-329955 36810321 PMC10176405

[26] Li C , Ma Y, Hua R, Zheng F, Xie W. Long-term physical activity participation trajectories were associated with subsequent cognitive decline, risk of dementia and all-cause mortality among adults aged ≥50 years: a population-based cohort study. Age Ageing. 2022;51:afac071. 10.1093/ageing/afac071 35348603

[27] Frank CC , Mundy LM, Smith J. Life course engagement in enriching activities: when and how does it matter for cognitive aging? Psychol Aging. 2023;38:263-276. 10.1037/pag0000744 37067480 PMC10238678

[28] Brown CJ , Jeon S, Ng YT, Lee S, Fingerman KL, Charles ST. Switching it up: activity diversity and cognitive functioning in later life. Psychol Aging. 2023;38:483-493. 10.1037/pag0000770 37535516 PMC10528947

[29] Carlson MC , Parisi JM, Xia J, et al. Lifestyle activities and memory: variety may be the spice of life. The Women’s Health and Aging Study II. J Int Neuropsychol Soc. 2012;18:286-294. 10.1017/S135561771100169X 22172155 PMC3508669

[30] Lee S , Charles ST, Almeida DM. Change is good for the brain: activity diversity and cognitive functioning across adulthood. The J Geron B Psychol Sci Soc Sci. 2021;76:1036-1048. 10.1093/geronb/gbaa020 PMC8200355 32025733

[31] Bielak AAM , Mogle JA, Sliwinski MJ. Two sides of the same coin? Association of variety and frequency of activity with cognition. Psychology and Aging. 2019;34:457-466. 10.1037/pag0000350 31070403

[32] Jackson JJ , Hill PL, Payne BR, Parisi JM, Stine-Morrow EAL. Linking openness to cognitive ability in older adulthood: the role of activity diversity. Aging Ment Health. 2020;24:1079-1087. 10.1080/13607863.2019.1655705 31446768 PMC7042045

[33] Jeon S , Lee S, Charles ST. Not just how much, but how many: overall and domain-specific activity variety and cognitive functioning in adulthood. J Geron B Psychol Sci Soc Sci. 2022;77:1229-1239. 10.1093/geronb/gbac053 PMC9255927 35291012

[34] Luo M , Moulder RG, Breitfelder LK, Röcke C. Daily activity diversity and daily working memory in community-dwelling older adults. Neuropsychology. 2023;37:181-193. 10.1037/neu0000878 36689393

[35] Peterson RL , Gilsanz P, George KM, et al. Differences in association of leisure time activities and cognition in a racially/ethnically diverse cohort of older adults: findings from the KHANDLE study. Alzheimers Dement (N Y). 2020;6:e12047. 10.1002/trc2.12047 32607410 PMC7317643

[36] Aartsen MJ , Cheval B, Sieber S, et al. Advantaged socioeconomic conditions in childhood are associated with higher cognitive functioning but stronger cognitive decline in older age. Proc Natl Acad Sci USA. 2019;116:5478-5486. 10.1073/pnas.1807679116 30804194 PMC6431198

[37] Mungas D , Gavett B, Fletcher E, Farias ST, DeCarli C, Reed B. Education amplifies brain atrophy effect on cognitive decline: implications for cognitive reserve. Neurobiol Aging. 2018;68:142-150. 10.1016/j.neurobiolaging.2018.04.002 29798764 PMC5993638

[38] Mroczek DK , Weston SJ, Graham EK, Willroth EC. Data overuse in aging research: emerging issues and potential solutions. Psychology and Aging. 2022;37:141-147. 10.1037/pag0000605 33914579 PMC8553804

[39] Brandt J , Spencer M, Folstein M. The telephone interview for cognitive status. Cogn Behav Neurol. 1988;1:111-117.

[40] Lachman ME , Tun PA. Cognitive testing in large-scale surveys: assessment by telephone. In: M Hofer, DF Alwin, eds. Handbook of Cognitive Aging: Interdisciplinary Perspectives. Sage Publications; 2008:506-523. 10.4135/9781412976589.n30

[41] Tun PA , Lachman ME. Telephone assessment of cognitive function in adulthood: the Brief Test of Adult Cognition by Telephone. Age and Ageing. 2006;35:629-632. 10.1093/­ageing/afl095 16943264

[42] Shannon CE. A mathematical theory of communication. Bell Syst. Tech. J. 1948;27:379-423. 10.1002/j.1538-7305.1948.tb01338.x

[43] Glymour MM. Commentary: modelling change in a causal framework. Int J Epidemiol. 2022;51:1615-1621. 10.1093/ije/dyac151 35900266 PMC9558068

[44] Gow AJ , Pattie A, Deary IJ. Lifecourse activity participation from early, mid, and later adulthood as determinants of cognitive aging: The Lothian Birth Cohort 1921. J Geron B Psychol Sci Soc Sci. 2017;72:25-37. 10.1093/geronb/gbw124 PMC5156497 27974473

[45] Vaportzis E , Niechcial MA, Gow AJ. A systematic literature review and meta-analysis of real-world interventions for cognitive ageing in healthy older adults. Ageing Res Rev. 2019;50:110-130. 10.1016/j.arr.2019.01.006 30707947

