# Supplementary material for: Does type or diversity of activities delay aging-related cognitive decline?
Source: Innov Aging. 2026 Feb 5;10(2):igaf133. doi: 10.1093/geroni/igaf133 (PMC12888382; doi:10.1093/geroni/igaf133)
Supplement: igaf133_Supplementary_Data [file igaf133_supplementary_data.zip › 29-Dec-2025_051339_innage_suppl_Glei,_Lee,_Brown,_Weinstein-REVISED.docx]

***Innovation in Aging* Supplementary Material: Glei, Lee, Brown, & Weinstein. Does Type or Diversity of Activities Delay Aging-Related Cognitive Decline?**

**Supplementary Methods**

**Section 1. Health and Retirement Study (HRS)**

The Health and Retirement Study (HRS) is sponsored by the National Institute on Aging (grant number NIA U01AG009740) and is conducted by the University of Michigan.^[dataset] 1^ HRS is a nationally representative longitudinal survey of persons above age 50 in the United States.^2^ In 1992, HRS sampled cohorts born in 1931-41 when they were approximately age 51-61. In subsequent waves, they sampled the cohorts born in 1923 or earlier (first interviewed in 1993), those born in 1924-30 and 1942-47 (first interviewed in 1998), cohorts born in 1948-53 (first interviewed in 2004), those born in 1954-59 (first interviewed in 2010-11), and cohorts born in 1960-65 (first interviewed in 2016). Each cohort was re-interviewed every two years. Thus, the sample represented the US population older than age 50 in 1998, 2004, 2010, and 2016. In addition to the targeted age-eligible respondent, HRS also interviewed the current spouse/partner (regardless of their age) at the time of the initial interview and all subsequent interviews.

The questions about cognitive and social activities came from the psychosocial and lifestyle self-administered questionnaire (SAQ). That SAQ has been administered since 2006 to an alternating random half of HRS core panel participants who completed the enhanced face-to-face interview, which also included physical measures and biomarkers.^3^ The questions about cognitive activities were included starting in 2008. Since 2016, the SAQ was administered only to self-respondents who were not institutionalized and completed their interview (through Section I, physical measures and biomarkers) in person.

[We have up to 7 obs for cohorts born < 1954; up to 5 obs for those born 1954-59; up to 3 obs for cohorts born 1960-65; (if we add 2022 wave) 1 obs for cohorts born 1966-71](we limited the HRS analyses to waves 2008- because 2008 was the first year for which the cognitive activity Qs were asked; physical activity Qs were available for 2004-2022; social contact Qs for 2004-2022; sport/social/other club for 2008-2022; social group meetings for 2004-2022].

**Section 2. Midlife in the United States (MIDUS) Study**

## **Original Cohort**

In 1995-96 (Wave 1), MIDUS targeted non-institutionalized, English-speaking adults aged 25-74^[[1]](#footnote-1)^ in the contiguous United States.^[dataset] 4^ National random digit dialing with oversampling of older people and men was used to select the main sample (*N*=3487), a sample of twin pairs (*N*=1914), and oversamples from five metropolitan areas in the U.S. (*N*=757). The study also included a random subsample of siblings of individuals in the main sample (*N*=950). The response rate for the phone interview ranged from 60% for the twin subsample to 70% for the main sample. Among those who completed the phone interview (*N*=7108), 6325 (89%) also completed a mail-in SAQ. In 2004-2005 (Wave 2), the MIDUS cohort was re-contacted: 4963 (75% of survivors from Wave 1) completed a follow-up telephone interview and 4041 (81% of those interviewed) completed the SAQ.^[dataset] 5^ Finally, the cohort was contacted again in 2013-2014 (Wave 3); 3294 (56% of survivors from Wave 1)[response rate computed in COREmortality.do] completed the telephone interview and 2732 (83% of those interviewed) completed the SAQ.^[dataset] 6^

## **Milwaukee Cohort**

At Wave 2, stratified area probability sampling was used to recruit a new oversample of African Americans in Milwaukee (aged 35-83), 592 of whom completed an in-person interview in 2005 (71% response rate), which was conducted using computer-assisted personal interviewing (CAPI); 416 (70% of those interviewed) completed the SAQ.^[dataset] 7^ At Wave 3, 389 of those respondents were re-interviewed via CAPI in 2016-17 (79% of survivors), and 327 (84% of those interviewed) completed the SAQ.^[dataset] 8^

## **Cognitive Assessments**

All those who completed the initial Wave 2 phone interview (including original and Milwaukee cohorts) were also eligible for the cognitive testing (fielded February 2004‒June 2006 in a separate phone interview). Cognitive assessments were completed by 4512 participants (81% of those who completed the initial phone interview) at Wave 2.^[dataset] 9^ At Wave 3,^[dataset] 10^ cognitive assessments (fielded July 2013–January 2018) were completed by 3291 participants (89% of those who completed the initial Wave 3 interview). Cognitive assessments were completed at both Waves 2 and 3 by 2940 respondents.

**Section 3: Measures of Cognitive Function**

#### **HRS**

The TICS has been administered to all HRS respondents since 1995/96. The four cognitive tests included immediate and delayed recall of ten nouns (both tests scored 0-10) and two tasks related to executive function: serial 7s subtraction (scored 0-5) and backwards counting (scored 0-2). HRS imputed missing data on the cognitive tests for self-respondents (but not interviews completed by proxy).^11^ Scores were summed to derive a measure of overall cognition (range 0-27) and then standardized (based on the baseline distribution).

#### **MIDUS**

The BTACT was administered separately from the initial MIDUS phone/CAPI interview. Cognitive tests related to episodic memory (i.e., immediate and delayed recall of 15 words based on the Rey Auditory Verbal Learning Test,^12,13^) and executive function (i.e., backward digit span,^14^ category verbal fluency,^15,16^ Stop and Go Switch Task,^17^ number series,^18,19^ and the 30 Seconds and Counting Task, a measure of processing speed^17^). We standardized the scores for each cognitive task at both waves based on the distribution at Wave 2. There were some extreme negative outliers on the Stop and Go Switch Task (i.e., values up to 27 SD below the mean) and also a few extreme positive outliers on the 30 Seconds and Counting Task (i.e., values more than 4.5 SD above the mean). Thus, for each cognitive test, we bottom- and top-coded extreme outliers (i.e., values less than the 25^th^ percentile minus 3 times the interquartile range (IQR) or greater than the 75^th^ percentile plus 3*IQR). Then, we averaged across all 7 standardized scores to obtain the composite score (Cronbach’s α = 0.76 for Wave 2 and 3). The final scores at both waves were re-standardized based on the distribution at Wave 2. Convergent and discriminant validity have been demonstrated among a subsample of individuals who were administered both the BTACT and an in-person comprehensive cognitive battery.^20^

# **Section 4: Measures of Contact Within Social Network**

## **HRS**

In HRS, respondents were asked about three types of contact (“meet up”, “speak on phone”, “write or email”) for three groups of network members (their children; other immediate family, for example, any brothers or sisters, parents, cousins or grandchildren; and friends) “not counting any who live with you.” All nine items were coded on a six-point scale. We recoded each item to the approximate frequency per month: never or less than once a year=0; once or twice a year=0.1; every few months=0.25; once or twice a month=1; once or twice a week=6; three or more times per week=12.

## **MIDUS**

MIDUS respondents were asked how often they were in contact (“including visits, phone calls, letters, or email”) with “members of your family…who do not live with you” and “any of your friends.” Both questions had nine response categories that ranged from “never” to “several times a day,” which we recoded into the approximate frequency of contact per month (never/hardly ever=0, less than once a month=0.5, about once a month=1, 2-3 times per month=2.5, about weekly=4, several times a week=8, about daily=30, several times a day=60).

# **Section 5: Measures of Social Group Participation**

## **HRS**

In HRS (see C:\Users\Dana Glei\Box\My Data\HRS\Data\ MIDUS-vs-HRS-Variables.xlsx)

HRS asked how often respondents “Go to a sport, social, or other club?” and “Attend meetings of non-religious organizations, such as political, community, or other interest groups?” Both questions had seven response categories, which we converted to the approximate frequency per month (Not in the last month=0, at least once a month=1, several times per month=2, Once a week=4, several times per week=8, daily=30).

Respondents were also asked how often they attend religious or spiritual services, with a five-point response scale, which we also converted to the approximate frequency per month (not at all=0, one or more times per year=0.25, two or three times a month=2, once a week=4, more than once a week=8).

Then, we summed across all three questions (sport/social/other clubs, meetings of non-religious organizations, religious/spiritual service attendance) to obtain the overall frequency per month (range=0 to 68).[Alternative version: Separate measures for religious attendance (range=0-8) and all other social group participation (range=0-60).]

## **MIDUS**

MIDUS asked how many times per month the respondent attended three types of social group meetings: unions or other professional groups; sports or social groups; any other groups not including any required by your job. They also asked about the frequency of attendance at religious or spiritual services, which had six response categories; we converted those responses to the approximate frequency per month (never=0, less than once a month=0.25, 1-3 times per month=2, once a week=4, a few times per week=8, daily=30). Then, we summed across the 4 items (range 0-79).[Alternative version: Separate measures for religious attendance (range=0-30) and all other social group participation (range=0-71).]

# **Section 6. Potential Confounders**

Our analysis includes 13 of the 14 modifiable risk factors for dementia highlighted in the Lancet report.^[[2]](#footnote-2)^ In addition to our key predictors (i.e., cognitive, physical, and social activity), those risk factors include other heath behaviors (i.e., smoking history, alcohol consumption), educational attainment, history of a head injury, and various measures of health status (i.e., hearing, vision, diabetes, hypertension, high cholesterol, obesity, and depression).

Many of those risk factors relate to cardiovascular health or can contribute to vascular disease—which, as noted in the introduction, is one of the indirect mechanisms through which physical activity can affect neurodegeneration. Specifically, smoking, alcohol consumption, hypertension, hypercholesterolemia, obesity, and diabetes may represent mediators through which activity influences cognition. However, vascular damage may have already occurred prior baseline and therefore, could limit activity at baseline (e.g., smoking, obesity, and chronic illness could inhibit physical activity as well as social interaction). Therefore, we adjusted for the baseline levels of these variables as well as history of heart problems, history of stroke, and physical limitations, which may indicate underlying health problems that influence both activity levels and cognition.

In addition, we adjusted for demographic characteristics (i.e., age, sex, race/ethnicity), marital status, employment status, and personality traits (i.e., extraversion, conscientiousness, neuroticism, openness, agreeableness),^21,22^ all of which were expected, based on prior literature, to affect both activity levels and cognition. Cognitive activities may be more feasible for some subgroups (e.g., younger people, those with higher educational attainment, those with better physical function), which is why it is important to control for those variables as potential confounders. Educational attainment was most strongly correlated with using a computer (*r*=0.40 in HRS; 0.38 in MIDUS), whereas the correlations with other cognitive activities were weaker (*r*=0.00–0.20 in HRS; -0.02–0.19 in MIDUS). Using a computer had the strongest inverse correlation with age (*r*= -0.30 in HRS; ‑0.22 in MIDUS) and physical limitations (*r*= -0.25 in HRS; -0.20 in MIDUS), but age was positively correlated with reading (*r*=0.19 in HRS; 0.24 in MIDUS) and playing word games (*r*=0.13 in HRS; 0.17 in MIDUS).

We categorized self-reported **alcohol consumption** as none, 1-7, 8-14, and 15+ drinks per week based on the Centers for Disease Control and Prevention definition for heavy drinking.^23^ **History of a head injury** is measured differently between the two surveys: in MIDUS, respondents were asked whether they have a history of a “*serious head injury*” (i.e., lifetime exposure), whereas in HRS they asked, “*Before you were 16 years old, did you have a blow to the head, a head injury or head trauma that was severe enough to require medical attention, to cause loss of consciousness or memory loss for a period of time?*”

In HRS, the questions measuring self-assessed **hearing** and **vision** were: “*Is your hearing excellent, very good, good, fair, or poor (using a hearing aid as usual)*?”; “*Is your eyesight excellent, very good, good, fair, or poor (using a glasses or corrective lenses as usual*?” In MIDUS, the corresponding questions were: “*Compared to other people your age, how would you rate your overall hearing?*”; “*Compared to other people your age, how would you rate your overall vision?*”

Blood pressure, cholesterol, and body mass index (BMI) were measured in HRS and MIDUS only for a subset of respondents. Therefore, we used self-reported measures. Hypertension was based on the following questions: (HRS) “*Has a doctor ever told you that you have high blood pressure or hypertension?*”; (MIDUS) “*In the past twelve months, have you experienced or been treated for high blood pressure or hypertension?*” The only self-reported information regarding cholesterol was medication use: (MIDUS) “*During the past 30 days, have you taken a prescription medicine for high cholesterol?*”; (HRS) “*Do you regularly take prescription medications to help* *lower your cholesterol?*” Obesity was categorized as BMI < 18.5 for underweight, 18.5-24.9 for normal weight, 25-29.9 for overweight, 30-34.9 for class I obesity, 35+ for class II/III obesity.

Depression was measured by the Composite International Diagnostic Interview Short Form (CIDI-SF) scale for major depression.^24^

For physical limitations, we included seven physical tasks that were reasonably comparable in both surveys: 1) lifting or carrying (MIDUS: “groceries”; HRS: “weights over 10 pounds, like a heavy bag of groceries”); 2) climbing several flights of stairs; 3) stooping, kneeling, bending, or crouching; 4) walking one block; 5) walking several blocks; 6) moderate activity (MIDUS: “moderate activity (e.g., bowling, vacuuming)”; HRS: “pulling or pushing large objects like a living room chair”); and 7) vigorous activity (MIDUS: “vigorous activity (e.g., running, lifting heavy objects)”; HRS: “running or jogging about a mile”). We counted the number of these tasks for which the respondent reported having any difficulty.

# **Section 7. Multiple Imputation**

Among the 86,567 HRS observations included in the analysis, activity diversity had the highest percentage of missing data (16%), followed by social contacts (12%), and cognitive activities (6%); missing data was less than 5% for other analysis variables. However, HRS already imputed data for cognition tests, as noted in the Measures section.

Among the 2,713 MIDUS respondents in the analysis, the variables with the most missing data were activity diversity (14%), taking medication for high cholesterol (13%), and social group participation (9%); missing data was less than 5% for other analysis variables.

We used the Stata user-written “ice” command to perform multiple imputation following standard practices.^25,26^ For the multiple imputation process, we used all the analysis variables as well as a few auxiliary measures (i.e., volunteer hours, number of physical limitations at Wave 3, self-assessed health status at Waves 2 and 3, and whether the respondent died by 12/31/2022 for MIDUS; volunteer work, self-assessed health status at baseline for HRS). We used an ordered logit model for imputation of ordinal variables (e.g., frequency of cognitive activities, religious attendance, hearing, vision, CIDI-SF depression scale, self-assessed health status). For continuous variables, departures from normality may result in implausible imputations when using the default draw method. Several variables had a skewed distribution (e.g., some cognitive tests, frequency of some types of physical activity, frequency of social group participation, alcohol consumption, body mass index). To ensure that imputed values were within the range of observed values, we used prediction matching for those variables as well as several others for which imputation generated out of range values (e.g., personality traits, physical limitations).

We performed five imputations. Then, we used the “mim” or “mi estimate” prefix command to re-estimate the model for each imputation and combine the five sets of estimates using Rubin’s rules.^27^

# **Section 8. Choice of Modeling Strategy**

## **Lagged Dependent Variable (LDV) Model**

Whether the analyst should control for the LDV has been debated for nearly 60 years, since Lord^28^ first noted that a model that adjusts for the baseline level can produce very different results from a model that does not (Lord’s Paradox). Recent work^29,30^ reveals that baseline adjustment yields misleading results in cases where the LDV is a potential mediator.

When the LDV is a potential mediator, the LDV model yields an estimate of the direct effect of the predictor on cognition at follow-up not mediated by cognition at baseline; that is, the effect on *exogenous* change in cognition.^30^ As Glymour^29^ explains, that estimand (i.e., the quantity that is being estimated) is of interest only when the LDV is a confounder.

In our case, there is likely to be a bidirectional relationship between activity engagement and cognition at baseline, as described in an example provided by Glymour^29^: cognitively-stimulating activities may slow memory decline, but someone with better memory at baseline is also more likely to engage in cognitively stimulating activities. In the presence of such feedback processes, the change score model is the best choice because it includes the feedback effects, whereas the direct effect estimated by the LDV model does not.

## **Lagged Independent Variable Model**

Tennant et al.^30^ advocates a different approach: using a lagged measure of the independent variable to predict a later measure of the outcome (i.e., total effect of baseline exposure on the outcome at follow-up). However, an analysis that uses lagged measures of activities to predict later cognitive function^22^ may also be biased by endogeneity (e.g., activity levels may be affected by earlier or concurrent levels of cognition, which are likely to be strongly correlated with later cognition).

As explained by Glymour,^29^ this modeling strategy conflates the determinants of cognition at the date of follow-up with the determinants of earlier cognitive function because it does not take into account change in the outcome that already occurred prior to follow-up. That it, physiological continuity (i.e., cognition earlier in life affects cognition later in life because is the same construct measured at two different periods) is component of that estimate, but it could not possibly be a causal effect of the predictor of interest because it was already present at baseline. Activity engagement at baseline cannot affect cognitive development that occurred prior to baseline. To understand what factors might alter the future rate of cognitive decline, we need to model the change in functioning.

## **Growth Curve Model**

When there are three or more repeated measurements of the outcome, a growth curve model is the most appropriate method. Tennant et al.^30^ and Glymour^29^ agree that growth curve modeling avoids the biases of the LDV model.

# **Supplementary Methods References**

1. University of Michigan. Health and Retirement Study, public use dataset. Produced and distributed by the University of Michigan with funding from the National Institute on Aging (grant number NIA U01AG009740). Published online 2024. https://hrs.isr.umich.edu/

2. Sonnega A, Faul JD, Ofstedal MB, Langa KM, Phillips JW, Weir DR. Cohort Profile: the Health and Retirement Study (HRS). *IntJEpidemiol*. 2014;43(2):576-585. doi:10.1093/ije/dyu067

3. Smith J, Ryan L, Larkina M, Sonnega A, Weir D. Psychosocial and Lifestyle Questionnaire 2006 - 2022: User Guide, Core Section LB. Published online 2023. Accessed January 30, 2025. https://hrs.isr.umich.edu/publications/biblio/12903

4. Brim OG, Baltes PB, Bumpass LL, et al. Midlife in the United States (MIDUS 1), 1995-1996. Published online 2020. doi:10.3886/ICPSR02760.v19

5. Ryff CD, Almeida DM, Ayanian JZ, et al. Midlife in the United States (MIDUS 2), 2004-2006. Published online 2021. doi:10.3886/ICPSR04652.v8

6. Ryff C, Almeida D, Ayanian J, et al. Midlife in the United States (MIDUS 3), 2013-2014. Published online 2019. doi:10.3886/ICPSR36346.v7

7. Ryff CD, Almeida DM, Ayanian JZ, et al. Midlife in the United States (MIDUS 2): Milwaukee African American Sample, 2005-2006. Published online 2024. doi:10.3886/ICPSR22840.v7

8. Ryff C, Almeida DM, Ayanian JS, et al. Midlife Development in the United States (MIDUS 3), Milwaukee African American Sample, 2016-2017.  Inter-university Consortium for Political and Social Research [distributor], Version 2 (2018-09-04). Published online 2018. doi:doi:10.3886/ICPSR37120.v2

9. Ryff C, Lachman ME. Midlife Development in the United States (MIDUS 2): Cognitive Project, 2004-2006.  Inter-university Consortium for Political and Social Research [distributor], Version 7 (2023-02-21). 2023;2020(February 11). doi:10.3886/ICPSR04652.v7

10. Ryff C, Lachman ME. Midlife Development in the United States (MIDUS 3): Cognitive Project, 2013-2017.  Inter-university Consortium for Political and Social Research [distributor], Version 3 (2023-08-17). 2023;2020(February 11). doi:10.3886/ICPSR04652.v7

11. McCammon RJ, Fisher GG, Hassan H, Faul JD, Rodgers WL, Weir DR. *Health and Retirement Study Imputation of Cognitive Functioning Measures, 1992 – 2020, Data Description*. Survey Research Center, University of Michigan; 2023. Accessed October 14, 2024. https://hrsdata.isr.umich.edu/sites/default/files/documentation/data-descriptions/1686605147/COGIMP9220_dd.pdf

12. Lezak MD. *Neuropsychological Assessment, Third Edition*. Oxford University Press; 1995.

13. Rey A. *L’Examen Clinique En Psychologie [The Clinical Examination in Psychology]*. Presses Universitaires de France; 1964.

14. Wechsler D. *Administration and Scoring Guide, WAIS – III, Wechsler Adult Intelligence Scale – Third Edition*. The Psychological Corporation, Harcourt Brace and Company; 1997.

15. Borkowski JG, Benton AL, Spreen O. Word fluency and brain damage. *Neuropsychologia*. 1967;5(2):135-140. doi:10.1016/0028-3932(67)90015-2

16. Tombaugh TN, Kozak J, Rees L. Normative Data Stratified by Age and Education for Two Measures of Verbal Fluency: FAS and Animal Naming. *Archives of Clinical Neuropsychology*. 1999;14(2):167-177.

17. Lachman ME, Tun PA. Cognitive testing in large-scale surveys: Assessment by telephone. In: Hofer M, Alwin DF, eds. *Handbook of Cognitive Aging: Interdisciplinary Perspectives*. Sage Publications; 2008:506-523. doi:10.4135/9781412976589.n30

18. Salthouse TA, Prill KA. Inferences about age impairments in inferential reasoning. *Psychol Aging*. 1987;2(1):43-51. doi:10.1037//0882-7974.2.1.43

19. Schaie KW. *Intellectual Development in Adulthood: The Seattle Longitudinal Study*. Cambridge University Pres; 1996.

20. Lachman ME, Agrigoroaei S, Tun PA, Weaver SL. Monitoring Cognitive Functioning: Psychometric Properties of the Brief Test of Adult Cognition by Telephone. *Assessment*. 2014;21(4):404-417. doi:10.1177/1073191113508807

21. Segel-Karpas D, Lachman ME. Social Contact and Cognitive Functioning: The Role of Personality. *The Journals of Gerontology: Series B*. 2018;73(6):974-984. doi:10.1093/geronb/gbw079

22. Stephan Y, Sutin AR, Luchetti M, Aschwanden D, Terracciano A. Physical, cognitive, and social activities as mediators between personality and cognition: evidence from four prospective samples. *Aging & Mental Health*. 2024;28(9):1294-1303. doi:10.1080/13607863.2024.2320135

23. Centers for Disease Control and Prevention. Alcohol Use and Your Health. Alcohol Use. June 11, 2024. Accessed October 14, 2024. https://www.cdc.gov/alcohol/about-alcohol-use/index.html

24. Kessler RC, Mickelson KD, Williams DR. The prevalence, distribution, and mental health correlates of perceived discrimination in the United States. *JHealth SocBehav*. 1999;40(3):208-230.

25. Rubin DB. Multiple imputation after 18+ years (with discussion). *J Am Stat Assoc*. 1996;91(Journal Article):473-489.

26. Schafer JL. Multiple imputation: a primer. *StatMethods MedRes*. 1999;8(1):3-15. doi:10.1177/096228029900800102

27. Royston P, Carlin JB, White IR. Multiple imputation of missing values: new features for mim. *Stata Journal*. 2009;9(2):252-264.

28. Lord FM. A paradox in the interpretation of group comparisons. *Psychological Bulletin*. 1967;68(5):304-305. doi:10.1037/h0025105

29. Glymour MM. Commentary: Modelling change in a causal framework. *International Journal of Epidemiology*. 2022;51(5):1615-1621. doi:10.1093/ije/dyac151

30. Tennant PWG, Arnold KF, Ellison GTH, Gilthorpe MS. Analyses of ‘change scores’ do not estimate causal effects in observational data. *Int J Epidemiol*. 2022;51(5):1604-1615. doi:10.1093/ije/dyab050

Supplementary Table 1. Information regarding sampling design, response rates, and analytic samples for each dataset

|  | **HRS 2008-2020** | **MIDUS Waves 2 (2004-05) & 3 (2013-14)** |
| --- | --- | --- |
| Survey Type | Longitudinal | Longitudinal |
| Population sampled | Persons born in 1890-1965 (and their spouses/partners) residing in households within the contiguous US (see Supplementary Methods Section 1 for more details) | Non-institutionalized, English-speaking residents of the contiguous US (see Supplementary Methods Section 2 for more details) |
| Age range | 18-109 ^a^ | 20-94 ^b^ |
| Sampling design | Multi-stage area probability sample with geographical stratification and clustering | At Wave 1, national random-digit dialing was used to select the main sample, a sample of twin pairs, and 5 metropolitan samples; in addition, a random subsample of siblings was selected from the siblings of individuals in the main sample. Stratified area probability sampling was used to select the Milwaukee sample added at Wave 2. |
| Subgroup(s) oversampled | Blacks  Latinx  Florida residents | Older persons  Men |
| Survey Modes | Face-to-Face or Phone ^c^ | Phone (initial interview and cognitive testing) and mail-in SAQ |
| Response Rate | For the core survey: ^d^  88.5% (2008)  81.0% (2010)  89.1% (2012)  87.1% (2014)  73.9% (2016)  74.4% (2018)  73.9% (2020)  Psychosocial SAQ ^e^  83.7% (2008)  73.1% (2010)  72.7% (2012)  77.8% (2014)  61.8% (2016)  64.7% (2018)  61.9% (2020) | At Wave 1 (% completed initial phone interview):  Main sample (70%)  Siblings (64%)  Twin pairs (60%)  Metro oversamples (N/A)  At Wave 2 (% completed initial interview):  Original cohort (75% of survivors from Wave 1)  Milwaukee sample (71%)  At Wave 3 (% completed initial interview):  Original cohort (56% of survivors from Wave 1)  Milwaukee sample (79% of survivors from Wave 2)  Cognitive testing (among those who completed initial interview at the specified wave):  Wave 2 (81%)  Wave 3 (89%)  (see Supplementary Methods Section 1 for more details) |
| Interviewed | 29,891 respondents ^f^  (132,333 observations) | 4,512 participants in cognitive testing at Wave 2 |
| **Excluded from analysis:** | | |
| Living in an institution | 2,832 observations ^g^ | -- |
| Interviewed by proxy | 5,297 observations | -- |
| Not age-eligible | 7,032 observations ^h^ | -- |
| Did not complete the SAQ | 16,450 observations ^i^ | 539 at Wave 2 |
| Other | 14,155 observations ^j^ | 1,260 at Wave 3^k^ |
| **Analytic sample** | **20,817 (respondents)**  **86,567 (observations)** | **2,713 respondents** |

Abbreviations: HRS, Health and Retirement Study; MIDUS, Midlife in the United States study; N/A, Not available; SAQ, Self-Administered Questionnaire.

^a^ The age-eligible respondents targeted by sampling comprised cohorts older than age 50 in 2010 and 2016, but HRS also interviewed the current spouse/partner at each wave. Thus, the age range of the full sample across Waves 2008-2020 was 18-109.

^b^ Although MIDUS targeted persons aged 25-74 at Wave 1 (1995-96), a few respondents were outside that age range (*N*=18 aged 20-24, *N*=5 aged 75). Participants were aged 28-85 by Wave 2 (2004-05) and aged 39-94 by Wave 3 (2013-14).

^c^ Most of the baseline interviews for HRS were conducted face-to-face, but subsequent interviews were often administered by telephone.^2^ Prior to 2004, follow-up interviews were conducted primarily by telephone although respondents over the age of 80 were offered a face-to-face interview. Since 2006, HRS has used a mixed-mode design for follow-up: about half of the interviews are conducted in-person, while the other half are administered via telephone. For supplemental studies, HRS also uses internet and self-administered mail surveys.

^d^ Source: <https://hrs.isr.umich.edu/documentation/survey-design/response-rates> (accessed 10/7/2024).

^e^ Source: Table 2 in the Core Section LB User Guide.^3^

^f^ The number of respondents ranged from 15,723 in 2020 to 22,034 in 2010.

^g^ The institutionalized population was not included in the initial sampling frame, but HRS followed those who became institutionalized after study entry. We excluded institutionalized respondents from HRS to enhance comparability with MIDUS.

^h^ For HRS, we restricted our analyses to the cohorts targeted by sampling (i.e., all waves 2008-2020 for those born before 1954; since 2010 for those born in 1954-59; since 2016 for those born in 1960-65), who were aged 50 or older at their initial interview.

^i^ We excluded 16,450 observations (for 6,240 respondents) who never completed the psychosocial and lifestyle SAQ.

^j^ For HRS, we also excluded 14,155 observations prior to the first wave in which the respondent completed the psychosocial SAQ (which serves at the baseline wave).

^k^ This group includes 1,040 who do not complete the main interview at Wave 3 and another 220 who did not participate in the Wave 3 cognitive testing.

Supplementary Table 2. Descriptive statistics for baseline measures, HRS and MIDUS

|  | **HRS** | **MIDUS** |
| --- | --- | --- |
| Age, mean (SD) | 65.7 (10.3) | 55.2 (11.3) |
| Female, % | 58.1 | 57.7 |
| Non-Hispanic White, % | 66.2 | 86.1 |
| Non-Hispanic Black, % | 17.8 | 9.4 |
| Non-Hispanic other races, % | 3.6 | 1.8 |
| Hispanic, % | 12.4 | 2.6 |
| Less than H.S. degree/GED, % | 17.6 | 4.6 |
| H.S. graduate/GED, % | 52.6 | 25.3 |
| Some college, % | 6.0 | 28.5 |
| College graduate, % | 14.1 | 24.0 |
| Graduate level degree, % | 9.7 | 17.6 |
| Married, % | 59.5 | 70.1 |
| Unmarried, % | 40.5 | 29.9 |
| Employed, % | 42.0 | 66.7 |
| Retired, % | 46.8 | 21.5 |
| Neither employed nor retired, % | 11.2 | 11.8 |
| Never smoked, % | 43.8 | 53.6 |
| Former smoker, % | 41.0 | 32.9 |
| Current smoker, % | 15.1 | 13.5 |
| Never drinks alcohol, % | 62.3 | 39.5 |
| 1-7 alcoholic drinks per week, % | 26.7 | 46.8 |
| 8-14 alcoholic drinks per week, % | 7.0 | 9.7 |
| 15 or more alcoholic drinks per week, % | 4.0 | 3.9 |
| History of a head injury,^a^ % | 9.4 | 3.3 |
| Self-assessed hearing (0-4=excellent), mean (SD) | 2.5 (1.1) | 2.5 (1.1) |
| Self-assessed vision (0-4=excellent), mean (SD) | 2.2 (1.0) | 2.3 (1.0) |
| Hypertension, % | 56.8 | 29.3 |
| Taking medication for high cholesterol, % | 41.8 | 25.6 |
| Underweight (BMI < 18.5), % | 1.2 | 0.8 |
| Normal (BMI 18.5-24.9), % | 26.1 | 30.7 |
| Overweight (BMI 25-29.9), % | 36.7 | 38.5 |
| Obese, Class I (BMI 30-34.9), % | 21.9 | 18.3 |
| Obese, Class II/III (BMI 35+), % | 14.1 | 11.6 |
| History of heart problems, % | 21.1 | 15.1 |
| History of stroke, % | 7.3 | 2.0 |
| Diabetes, % | 21.8 | 8.5 |
| Physical limitations (0-7), mean (SD) | 2.7 (2.2) | 2.5 (2.4) |
| Depressive symptoms (0-7), mean (SD) | 0.5 (1.6) | 0.6 (1.7) |
| Extraversion (1-4), mean (SD) | 3.2 (0.6) | 3.1 (0.6) |
| Conscientiousness (1-4), mean (SD) | 3.4 (0.5) | 3.4 (0.5) |
| Neuroticism (1-4), mean (SD) | 2.0 (0.6) | 2.1 (0.6) |
| Openness (1-4), mean (SD) | 2.9 (0.6) | 2.9 (0.5) |
| Agreeableness (1-4), mean (SD) | 3.5 (0.5) | 3.5 (0.5) |
| Cognitive activities (0-180),^b^ mean (SD) | 45.5 (30.4) | 52.5 (27.3) |
| Physical activity (0-90),^b,c^ mean (SD) | 18.3 (15.6) | 29.9 (16.4) |
| Contact with family and friends (0-120),^b,d^ mean (SD) | 30.3 (19.3) | 36.2 (31.2) |
| Social group participation (0-79),^b,e^ mean (SD) | 4.3 (5.5) | 6.7 (7.9) |
| Activity diversity (0-1), mean (SD) | 0.7 (0.2) | 0.8 (0.1) |
| Cognition,^f^ mean (SD) | 0.0 (1.0) | 0.0 (1.0) |
| Number of respondents | 20,817 | 2,713 |

*Note*. BMI = Body Mass Index. Baseline was defined as the first available observation for HRS (2008 for cohorts born before 1954; 2010 for those born in 1954–59; 2016 for those born in 1960–65) and 2004–06 for MIDUS.

^a^ In MIDUS, this variable reflects lifetime exposure (i.e., prior to baseline) to a serious head injury, whereas HRS asked only about head injuries prior to age 16.

^b^ Frequency per month.

^c^ The maximum was 90 in HRS and 72 in MIDUS.

^d^ The maximum was 108 in HRS and 120 in MIDUS.

^e^ The maximum was 68 in HRS and 79 in MIDUS.

^f^ Cognition was standardized based on the distribution at baseline within each dataset.

Supplementary Table 3. Coefficients (& 95% confidence intervals) from growth curve models predicting age trajectory of cognition, HRS 2008-2020 (*N*=86,567 observations)

|  | (1) | (2) | (3) |
| --- | --- | --- | --- |
| Age (decades after 65)^a^ | -0.157*** | -0.160*** | -0.174*** |
|  | (-0.173, -0.140) | (-0.203, -0.117) | (-0.218, -0.130) |
| Age (decades after 65) squared | -0.137*** | -0.104*** | -0.104*** |
|  | (-0.144, -0.129) | (-0.131, -0.077) | (-0.131, -0.077) |
| Effects on cognition at age 65 |  |  |  |
| Cognitive activity^b,c^ | 0.307*** | 0.096*** |  |
|  | (0.293, 0.322) | (0.084, 0.109) |  |
| Physical activity^b,c^ | 0.079*** | 0.010 |  |
|  | (0.063, 0.096) | (-0.003, 0.022) |  |
| Social contact^b,c^ | -0.011 | 0.010 |  |
|  | (-0.023, 0.002) | (-0.003, 0.023) |  |
| Social group participation^b,c^ | -0.028** | -0.016* |  |
|  | (-0.046, -0.010) | (-0.030, -0.003) |  |
| Activity diversity^c^ |  |  | 0.064*** |
|  |  |  | (0.053, 0.076) |
| Female |  | 0.193*** | 0.217*** |
|  |  | (0.170, 0.217) | (0.192, 0.242) |
| Non-Hispanic White (*omitted*) |  | *1.00* | *1.00* |
| Non-Hispanic Black |  | -0.436*** | -0.480*** |
|  |  | (-0.474, -0.398) | (-0.519, -0.440) |
| Non-Hispanic other races |  | -0.226*** | -0.251*** |
|  |  | (-0.295, -0.156) | (-0.321, -0.181) |
| Hispanic |  | -0.214*** | -0.249*** |
|  |  | (-0.270, -0.159) | (-0.304, -0.194) |
| Less than H.S. degree/GED |  | -0.478*** | -0.498*** |
|  |  | (-0.514, -0.442) | (-0.534, -0.462) |
| H.S. graduate/GED (*omitted*) |  | *1.00* | *1.00* |
| Some college |  | 0.109*** | 0.126*** |
|  |  | (0.070, 0.148) | (0.087, 0.166) |
| College graduate |  | 0.292*** | 0.316*** |
|  |  | (0.257, 0.327) | (0.281, 0.351) |
| Graduate level degree |  | 0.390*** | 0.431*** |
|  |  | (0.352, 0.429) | (0.392, 0.470) |
| Married (*omitted*) |  | *1.00* | *1.00* |
| Unmarried |  | -0.064*** | -0.061*** |
|  |  | (-0.089, -0.040) | (-0.086, -0.037) |
| Employed (*omitted*) |  | *1.00* | *1.00* |
| Retired |  | -0.095*** | -0.100*** |
|  |  | (-0.124, -0.067) | (-0.129, -0.072) |
| Neither employed nor retired |  | -0.160*** | -0.165*** |
|  |  | (-0.198, -0.122) | (-0.203, -0.127) |
| Never smoked (*omitted*) |  | *1.00* | *1.00* |
| Former smoker |  | 0.004 | 0.012 |
|  |  | (-0.021, 0.029) | (-0.013, 0.038) |
| Current smoker |  | -0.088*** | -0.070*** |
|  |  | (-0.127, -0.049) | (-0.110, -0.030) |
| Never drinks alcohol *(omitted)* |  | 1.00 | 1.00 |
| 1-7 drinks per week |  | 0.068*** | 0.076*** |
|  |  | (0.043, 0.093) | (0.051, 0.102) |
| 8-14 drinks per week |  | 0.070** | 0.080*** |
|  |  | (0.027, 0.114) | (0.037, 0.122) |
| 15 or more drinks per week |  | 0.071* | 0.082** |
|  |  | (0.013, 0.130) | (0.023, 0.141) |
| History of a head injury |  | 0.010 | 0.016 |
|  |  | (-0.028, 0.048) | (-0.022, 0.053) |
| Self-assessed hearing^c^ |  | 0.029*** | 0.030*** |
|  |  | (0.017, 0.041) | (0.018, 0.041) |
| Self-assessed vision^c^ |  | 0.061*** | 0.064*** |
|  |  | (0.048, 0.073) | (0.051, 0.077) |
| Hypertension |  | -0.056*** | -0.056*** |
|  |  | (-0.081, -0.030) | (-0.082, -0.031) |
| Medication for high cholesterol |  | -0.023 | -0.023 |
|  |  | (-0.049, 0.003) | (-0.049, 0.003) |
| Underweight (BMI < 18.5) |  | -0.120* | -0.110* |
|  |  | (-0.228, -0.012) | (-0.218, -0.003) |
| Normal (BMI 18.5-24.9) *(omitted)* |  | 1.00 | 1.00 |
| Overweight (BMI 25-29.9) |  | 0.054*** | 0.056*** |
|  |  | (0.028, 0.081) | (0.029, 0.083) |
| Obese, Class I (BMI 30-34.9) |  | 0.080*** | 0.089*** |
|  |  | (0.047, 0.113) | (0.055, 0.122) |
| Obese, Class II/III (BMI 35+) |  | 0.117*** | 0.135*** |
|  |  | (0.081, 0.152) | (0.098, 0.172) |
| Heart problems |  | 0.011 | 0.013 |
|  |  | (-0.017, 0.040) | (-0.016, 0.041) |
| Stroke |  | -0.172*** | -0.165*** |
|  |  | (-0.226, -0.118) | (-0.219, -0.111) |
| Diabetes |  | -0.052*** | -0.052*** |
|  |  | (-0.082, -0.023) | (-0.082, -0.023) |
| Physical limitations^c^ |  | -0.066*** | -0.061*** |
|  |  | (-0.080, -0.052) | (-0.075, -0.047) |
| Depressive symptoms^c^ |  | -0.005 | -0.003 |
|  |  | (-0.016, 0.007) | (-0.015, 0.009) |
| Extraversion^c^ |  | -0.061*** | -0.072*** |
|  |  | (-0.076, -0.045) | (-0.087, -0.056) |
| Conscientiousness^c^ |  | 0.052*** | 0.053*** |
|  |  | (0.038, 0.065) | (0.040, 0.067) |
| Neuroticism^c^ |  | -0.030*** | -0.032*** |
|  |  | (-0.043, -0.016) | (-0.045, -0.018) |
| Openness^c^ |  | 0.043*** | 0.061*** |
|  |  | (0.030, 0.056) | (0.048, 0.074) |
| Agreeableness^c^ |  | 0.026*** | 0.025*** |
|  |  | (0.011, 0.040) | (0.011, 0.039) |
| Effects on rate of cognitive decline |  |  |  |
| Cognitive activity^c^ × age | 0.016* | 0.018** |  |
|  | (0.003, 0.029) | (0.007, 0.030) |  |
| Cognitive activity^c^ × age^2^ | -0.002 | 0.004 |  |
|  | (-0.010, 0.005) | (-0.004, 0.011) |  |
| Physical activity^c^ × age | 0.004 | 0.000 |  |
|  | (-0.008, 0.015) | (-0.010, 0.011) |  |
| Physical activity^c^ × age^2^ | -0.005 | -0.001 |  |
|  | (-0.013, 0.002) | (-0.009, 0.006) |  |
| Social contact^c^ × age | 0.020** | 0.015* |  |
|  | (0.007, 0.033) | (0.003, 0.027) |  |
| Social contact^c^ × age^2^ | -0.009* | -0.006 |  |
|  | (-0.017, -0.001) | (-0.014, 0.002) |  |
| Group participation^c^ × age | 0.013 | 0.005 |  |
|  | (-0.000, 0.025) | (-0.007, 0.016) |  |
| Group participation^c^ × age^2^ | 0.008 | 0.007 |  |
|  | (-0.000, 0.016) | (-0.000, 0.015) |  |
| Activity diversity^c^ × age |  |  | 0.018** |
|  |  |  | (0.006, 0.030) |
| Activity diversity^c^ × age^2^ |  |  | -0.009** |
|  |  |  | (-0.015, -0.003) |
| Female × age |  | 0.038*** | 0.050*** |
|  |  | (0.017, 0.059) | (0.030, 0.071) |
| Female × age^2^ |  | -0.030*** | -0.031*** |
|  |  | (-0.043, -0.017) | (-0.044, -0.018) |
| Non-Hispanic Black × age |  | 0.017 | 0.010 |
|  |  | (-0.017, 0.051) | (-0.023, 0.044) |
| Non-Hispanic Black × age^2^ |  | -0.011 | -0.012 |
|  |  | (-0.031, 0.009) | (-0.032, 0.009) |
| Non-Hispanic other races × age |  | 0.030 | 0.018 |
|  |  | (-0.040, 0.099) | (-0.051, 0.088) |
| Non-Hispanic other races × age^2^ |  | -0.021 | -0.020 |
|  |  | (-0.071, 0.028) | (-0.069, 0.029) |
| Hispanic × age |  | 0.075*** | 0.073*** |
|  |  | (0.040, 0.110) | (0.038, 0.107) |
| Hispanic × age^2^ |  | -0.026* | -0.027* |
|  |  | (-0.050, -0.002) | (-0.051, -0.004) |
| Less than H.S. degree/GED × age |  | -0.038 | -0.044* |
|  |  | (-0.077, 0.002) | (-0.083, -0.004) |
| Less than H.S. degree/GED × age^2^ |  | 0.017 | 0.015 |
|  |  | (-0.004, 0.037) | (-0.005, 0.035) |
| Some college × age |  | -0.034 | -0.029 |
|  |  | (-0.078, 0.009) | (-0.073, 0.014) |
| Some college × age^2^ |  | 0.019 | 0.016 |
|  |  | (-0.007, 0.045) | (-0.010, 0.042) |
| College graduate × age |  | -0.015 | -0.010 |
|  |  | (-0.042, 0.013) | (-0.038, 0.017) |
| College graduate × age^2^ |  | -0.016 | -0.014 |
|  |  | (-0.035, 0.003) | (-0.033, 0.006) |
| Graduate level degree × age |  | -0.025 | -0.017 |
|  |  | (-0.059, 0.009) | (-0.051, 0.016) |
| Graduate level degree × age^2^ |  | -0.013 | -0.015 |
|  |  | (-0.035, 0.008) | (-0.037, 0.008) |
| Unmarried × age |  | 0.034** | 0.032** |
|  |  | (0.010, 0.059) | (0.008, 0.057) |
| Unmarried × age^2^ |  | -0.005 | -0.006 |
|  |  | (-0.021, 0.010) | (-0.021, 0.009) |
| Retired × age |  | 0.038* | 0.035* |
|  |  | (0.007, 0.069) | (0.003, 0.066) |
| Retired × age^2^ |  | -0.036*** | -0.034*** |
|  |  | (-0.055, -0.018) | (-0.052, -0.015) |
| Neither employed nor retired × age |  | -0.004 | -0.005 |
|  |  | (-0.044, 0.036) | (-0.046, 0.036) |
| Neither employed nor retired × age^2^ |  | -0.013 | -0.013 |
|  |  | (-0.040, 0.015) | (-0.041, 0.014) |
| Former smoker × age |  | -0.030* | -0.024 |
|  |  | (-0.054, -0.005) | (-0.048, 0.001) |
| Former smoker × age^2^ |  | 0.001 | -0.000 |
|  |  | (-0.013, 0.015) | (-0.014, 0.014) |
| Current smoker × age |  | -0.042* | -0.033 |
|  |  | (-0.075, -0.008) | (-0.067, 0.001) |
| Current smoker × age^2^ |  | 0.004 | -0.002 |
|  |  | (-0.025, 0.033) | (-0.031, 0.027) |
| 1-7 drinks/week × age |  | 0.012 | 0.015 |
|  |  | (-0.011, 0.035) | (-0.008, 0.038) |
| 1-7 drinks/week × age^2^ |  | 0.001 | 0.001 |
|  |  | (-0.017, 0.019) | (-0.017, 0.019) |
| 8-14 drinks/week × age |  | -0.011 | -0.005 |
|  |  | (-0.050, 0.028) | (-0.044, 0.034) |
| 8-14 drinks/week × age^2^ |  | 0.027 | 0.027* |
|  |  | (-0.001, 0.054) | (0.000, 0.055) |
| 15+ drinks/week × age |  | -0.027 | -0.024 |
|  |  | (-0.078, 0.024) | (-0.077, 0.029) |
| 15+ drinks/week × age^2^ |  | -0.021 | -0.022 |
|  |  | (-0.067, 0.025) | (-0.068, 0.024) |
| Head injury × age |  | -0.025 | -0.018 |
|  |  | (-0.130, 0.079) | (-0.123, 0.087) |
| Head injury × age^2^ |  | 0.020 | 0.021 |
|  |  | (-0.034, 0.075) | (-0.033, 0.074) |
| Hearing^c^ × age |  | 0.004 | 0.006 |
|  |  | (-0.022, 0.031) | (-0.021, 0.034) |
| Hearing^c^ × age^2^ |  | -0.001 | 0.000 |
|  |  | (-0.017, 0.014) | (-0.016, 0.016) |
| Vision^c^ × age |  | 0.002 | 0.007 |
|  |  | (-0.031, 0.034) | (-0.024, 0.039) |
| Vision^c^ × age^2^ |  | 0.007 | 0.007 |
|  |  | (-0.016, 0.029) | (-0.016, 0.030) |
| Hypertension × age |  | -0.018 | -0.010 |
|  |  | (-0.057, 0.022) | (-0.049, 0.029) |
| Hypertension × age^2^ |  | -0.002 | -0.004 |
|  |  | (-0.025, 0.022) | (-0.028, 0.019) |
| Cholesterol medication × age |  | -0.033 | -0.033 |
|  |  | (-0.067, 0.000) | (-0.066, 0.000) |
| Cholesterol medication × age^2^ |  | 0.014 | 0.014 |
|  |  | (-0.008, 0.035) | (-0.008, 0.035) |
| Underweight × age |  | -0.001 | -0.000 |
|  |  | (-0.012, 0.011) | (-0.011, 0.011) |
| Underweight × age^2^ |  | -0.002 | -0.002 |
|  |  | (-0.008, 0.004) | (-0.008, 0.004) |
| Overweight × age |  | -0.010 | -0.009 |
|  |  | (-0.022, 0.001) | (-0.020, 0.002) |
| Overweight × age^2^ |  | -0.005 | -0.004 |
|  |  | (-0.012, 0.002) | (-0.011, 0.002) |
| Obese, Class I × age |  | 0.032** | 0.032** |
|  |  | (0.011, 0.053) | (0.011, 0.053) |
| Obese, Class I × age^2^ |  | 0.005 | 0.007 |
|  |  | (-0.009, 0.020) | (-0.007, 0.022) |
| Obese, Class II/III × age |  | 0.010 | 0.009 |
|  |  | (-0.019, 0.039) | (-0.020, 0.038) |
| Obese, Class II/III × age^2^ |  | -0.002 | -0.001 |
|  |  | (-0.018, 0.014) | (-0.017, 0.014) |
| Heart problems × age |  | -0.031 | -0.031 |
|  |  | (-0.066, 0.004) | (-0.065, 0.004) |
| Heart problems × age^2^ |  | 0.018* | 0.017 |
|  |  | (0.000, 0.035) | (-0.000, 0.035) |
| Stroke × age |  | -0.027 | -0.027 |
|  |  | (-0.089, 0.034) | (-0.090, 0.036) |
| Stroke × age^2^ |  | 0.020 | 0.018 |
|  |  | (-0.008, 0.047) | (-0.011, 0.046) |
| Diabetes × age |  | -0.056*** | -0.057*** |
|  |  | (-0.086, -0.026) | (-0.087, -0.027) |
| Diabetes × age^2^ |  | 0.008 | 0.007 |
|  |  | (-0.011, 0.026) | (-0.012, 0.026) |
| Physical limitations^c^ × age |  | -0.012 | -0.010 |
|  |  | (-0.027, 0.003) | (-0.025, 0.005) |
| Physical limitations^c^ × age^2^ |  | 0.010** | 0.009* |
|  |  | (0.004, 0.017) | (0.002, 0.016) |
| Depressive symptoms^c^ × age |  | -0.013* | -0.014* |
|  |  | (-0.025, -0.002) | (-0.026, -0.003) |
| Depressive symptoms^c^ × age^2^ |  | 0.007 | 0.006 |
|  |  | (-0.001, 0.015) | (-0.002, 0.014) |
| Extraversion^c^ × age |  | 0.003 | 0.002 |
|  |  | (-0.012, 0.018) | (-0.013, 0.017) |
| Extraversion^c^ × age^2^ |  | -0.007 | -0.006 |
|  |  | (-0.016, 0.003) | (-0.015, 0.003) |
| Conscientiousness^c^ × age |  | 0.013* | 0.010 |
|  |  | (0.000, 0.026) | (-0.003, 0.022) |
| Conscientiousness^c^ × age^2^ |  | -0.001 | -0.000 |
|  |  | (-0.008, 0.007) | (-0.007, 0.007) |
| Neuroticism^c^ × age |  | -0.007 | -0.008 |
|  |  | (-0.019, 0.006) | (-0.021, 0.004) |
| Neuroticism^c^ × age^2^ |  | -0.005 | -0.006 |
|  |  | (-0.013, 0.003) | (-0.014, 0.003) |
| Openness^c^ × age |  | -0.003 | 0.001 |
|  |  | (-0.016, 0.010) | (-0.012, 0.014) |
| Openness^c^ × age^2^ |  | -0.000 | -0.001 |
|  |  | (-0.008, 0.007) | (-0.008, 0.007) |
| Agreeableness^c^ × age |  | -0.010 | -0.009 |
|  |  | (-0.024, 0.004) | (-0.023, 0.005) |
| Agreeableness^c^ × age^2^ |  | 0.002 | 0.001 |
|  |  | (-0.006, 0.010) | (-0.006, 0.009) |
| Constant | 0.180*** | 0.232*** | 0.212*** |
|  | (0.156, 0.203) | (0.192, 0.271) | (0.172, 0.252) |

Abbreviations: BMI = Body Mass Index

^a^ Age was measured in decades after age 65: $\left( \text{age}\boldsymbol{-65} \right)/\boldsymbol{10}$); thus, the coefficient represents the effect per 10 years of aging.

^b^ Frequency per month.

^c^ Standardized so that coefficient represents the effect per SD.

Supplementary Table 4. Coefficients (& 95% confidence intervals) from cross-sectional regression of cognition on activities (both measured at Wave 2), MIDUS (*N*=2713)

|  | (1) | (2) | (3) |
| --- | --- | --- | --- |
| Age (decades after 65)^a^ | -0.374*** | -0.348*** | -0.347*** |
|  | (-0.423, -0.325) | (-0.404, -0.291) | (-0.404, -0.290) |
| Age (decades after 65) squared | -0.030* | -0.031** | -0.036** |
|  | (-0.054, -0.006) | (-0.054, -0.009) | (-0.059, -0.014) |
| Cognitive activity^b,c^ | 0.285*** | 0.165*** |  |
|  | (0.250, 0.319) | (0.132, 0.198) |  |
| Physical activity^b,c^ | 0.090*** | 0.036* |  |
|  | (0.054, 0.125) | (0.003, 0.070) |  |
| Social contact^b,c^ | -0.037* | -0.004 |  |
|  | (-0.073, -0.001) | (-0.037, 0.029) |  |
| Social group participation^b,c^ | 0.020 | 0.007 |  |
|  | (-0.015, 0.054) | (-0.023, 0.037) |  |
| Activity diversity^c^ |  |  | 0.059*** |
|  |  |  | (0.026, 0.092) |
| Female |  | 0.112** | 0.137*** |
|  |  | (0.041, 0.183) | (0.066, 0.209) |
| Non-Hispanic White (*omitted*) |  | *1.00* | *1.00* |
| Non-Hispanic Black |  | -0.725*** | -0.745*** |
|  |  | (-0.842, -0.609) | (-0.860, -0.630) |
| Non-Hispanic other races |  | -0.355** | -0.365** |
|  |  | (-0.588, -0.121) | (-0.607, -0.123) |
| Hispanic |  | -0.204 | -0.205 |
|  |  | (-0.413, 0.005) | (-0.426, 0.017) |
| Less than H.S. degree/GED |  | -0.382*** | -0.371*** |
|  |  | (-0.534, -0.231) | (-0.527, -0.215) |
| H.S. graduate/GED (*omitted*) |  | *1.00* | *1.00* |
| Some college |  | 0.187*** | 0.224*** |
|  |  | (0.101, 0.272) | (0.138, 0.311) |
| College graduate |  | 0.463*** | 0.548*** |
|  |  | (0.369, 0.557) | (0.455, 0.640) |
| Graduate level degree |  | 0.599*** | 0.711*** |
|  |  | (0.497, 0.700) | (0.609, 0.813) |
| Married (*omitted*) |  | *1.00* | *1.00* |
| Unmarried |  | -0.051 | -0.063 |
|  |  | (-0.123, 0.020) | (-0.135, 0.009) |
| Employed (*omitted*) |  | *1.00* | *1.00* |
| Retired |  | -0.040 | -0.063 |
|  |  | (-0.134, 0.053) | (-0.159, 0.033) |
| Neither employed nor retired |  | 0.003 | -0.018 |
|  |  | (-0.101, 0.107) | (-0.123, 0.088) |
| Never smoked (*omitted*) |  | *1.00* | *1.00* |
| Former smoker |  | 0.009 | 0.021 |
|  |  | (-0.062, 0.081) | (-0.052, 0.094) |
| Current smoker |  | 0.002 | 0.008 |
|  |  | (-0.100, 0.103) | (-0.096, 0.111) |
| Never drinks alcohol *(omitted)* |  | *1.00* | *1.00* |
| 1-7 drinks per week |  | 0.087* | 0.116*** |
|  |  | (0.019, 0.154) | (0.048, 0.184) |
| 8-14 drinks per week |  | 0.092 | 0.121* |
|  |  | (-0.018, 0.202) | (0.010, 0.232) |
| 15 or more drinks per week |  | 0.173* | 0.186* |
|  |  | (0.013, 0.333) | (0.021, 0.351) |
| History of a head injury |  | -0.082 | -0.104 |
|  |  | (-0.257, 0.093) | (-0.282, 0.073) |
| Self-assessed hearing^c^ |  | 0.025 | 0.028 |
|  |  | (-0.009, 0.060) | (-0.007, 0.063) |
| Self-assessed vision^c^ |  | 0.041* | 0.045* |
|  |  | (0.006, 0.076) | (0.009, 0.081) |
| Hypertension |  | -0.041 | -0.037 |
|  |  | (-0.115, 0.033) | (-0.112, 0.038) |
| Medication for high cholesterol |  | 0.003 | 0.010 |
|  |  | (-0.075, 0.082) | (-0.070, 0.090) |
| Underweight (BMI < 18.5) |  | 0.220 | 0.224 |
|  |  | (-0.230, 0.669) | (-0.229, 0.676) |
| Normal (BMI 18.5-24.9) *(omitted)* |  | *1.00* | *1.00* |
| Overweight (BMI 25-29.9) |  | 0.011 | 0.024 |
|  |  | (-0.066, 0.087) | (-0.054, 0.103) |
| Obese, Class I (BMI 30-34.9) |  | -0.051 | -0.024 |
|  |  | (-0.148, 0.046) | (-0.122, 0.074) |
| Obese, Class II/III (BMI 35+) |  | -0.056 | -0.026 |
|  |  | (-0.173, 0.062) | (-0.145, 0.092) |
| Heart problems |  | -0.014 | -0.011 |
|  |  | (-0.101, 0.072) | (-0.099, 0.076) |
| Stroke |  | -0.358*** | -0.368*** |
|  |  | (-0.554, -0.163) | (-0.573, -0.163) |
| Diabetes |  | -0.109 | -0.101 |
|  |  | (-0.225, 0.006) | (-0.220, 0.019) |
| Physical limitations^c^ |  | -0.008 | -0.013 |
|  |  | (-0.046, 0.029) | (-0.051, 0.025) |
| Depressive symptoms^c^ |  | -0.029 | -0.020 |
|  |  | (-0.060, 0.001) | (-0.052, 0.013) |
| Extraversion^c^ |  | -0.018 | -0.030 |
|  |  | (-0.059, 0.023) | (-0.070, 0.011) |
| Conscientiousness^c^ |  | 0.005 | 0.011 |
|  |  | (-0.029, 0.038) | (-0.023, 0.046) |
| Neuroticism^c^ |  | -0.051** | -0.054** |
|  |  | (-0.085, -0.017) | (-0.089, -0.019) |
| Openness^c^ |  | 0.015 | 0.051* |
|  |  | (-0.025, 0.056) | (0.011, 0.091) |
| Agreeableness^c^ |  | 0.001 | -0.007 |
|  |  | (-0.037, 0.038) | (-0.045, 0.031) |
| Constant | -0.299*** | -0.499*** | -0.579*** |
|  | (-0.346, -0.252) | (-0.623, -0.375) | (-0.704, -0.453) |

Abbreviations: BMI = Body Mass Index

^a^ Age was measured in decades after age 65: $\left( \text{age}\boldsymbol{-65} \right)/\boldsymbol{10}$); thus, the coefficient represents the effect per 10 years of aging.

^b^ Frequency per month.

^c^ Standardized so that coefficient represents the effect per SD.

Supplementary Table 5. Coefficients (& 95% confidence intervals) from prospective models using activities (at Wave 2) to predict subsequent changes in cognition (between Waves 2 and 3),^a^ MIDUS (*N*=2713)

|  | (1) | (2) | (3) |
| --- | --- | --- | --- |
| Age (decades after 65)^b^ | -0.232*** | -0.196*** | -0.198*** |
|  | (-0.280, -0.184) | (-0.258, -0.135) | (-0.260, -0.137) |
| Age (decades after 65) squared | -0.032** | -0.024* | -0.024* |
|  | (-0.053, -0.012) | (-0.046, -0.001) | (-0.046, -0.001) |
| Cognitive activity^c,d^ | -0.018 | -0.013 |  |
|  | (-0.048, 0.012) | (-0.047, 0.020) |  |
| Physical activity^c,d^ | 0.004 | -0.000 |  |
|  | (-0.025, 0.034) | (-0.031, 0.031) |  |
| Social contact^c,d^ | 0.016 | 0.017 |  |
|  | (-0.013, 0.046) | (-0.015, 0.049) |  |
| Social group participation^c,d^ | -0.007 | -0.015 |  |
|  | (-0.038, 0.023) | (-0.048, 0.017) |  |
| Activity diversity^d^ |  |  | 0.000 |
|  |  |  | (-0.030, 0.031) |
| Female |  | 0.057 | 0.057 |
|  |  | (-0.012, 0.126) | (-0.012, 0.125) |
| Non-Hispanic White (*omitted*) |  | *1.00* | *1.00* |
| Non-Hispanic Black |  | 0.042 | 0.036 |
|  |  | (-0.070, 0.153) | (-0.075, 0.147) |
| Non-Hispanic other races |  | 0.162 | 0.164 |
|  |  | (-0.088, 0.411) | (-0.085, 0.413) |
| Hispanic |  | 0.008 | 0.011 |
|  |  | (-0.193, 0.209) | (-0.190, 0.211) |
| Less than H.S. degree/GED |  | 0.109 | 0.114 |
|  |  | (-0.020, 0.238) | (-0.016, 0.244) |
| H.S. graduate/GED (*omitted*) |  | *1.00* | *1.00* |
| Some college |  | -0.011 | -0.016 |
|  |  | (-0.092, 0.069) | (-0.096, 0.064) |
| College graduate |  | -0.028 | -0.039 |
|  |  | (-0.125, 0.069) | (-0.133, 0.055) |
| Graduate level degree |  | -0.054 | -0.069 |
|  |  | (-0.160, 0.052) | (-0.170, 0.032) |
| Married (*omitted*) |  | *1.00* | *1.00* |
| Unmarried |  | -0.027 | -0.022 |
|  |  | (-0.101, 0.048) | (-0.096, 0.052) |
| Employed (*omitted*) |  | *1.00* | *1.00* |
| Retired |  | -0.122* | -0.123* |
|  |  | (-0.220, -0.025) | (-0.220, -0.026) |
| Neither employed nor retired |  | -0.142** | -0.141** |
|  |  | (-0.238, -0.045) | (-0.236, -0.045) |
| Never smoked (*omitted*) |  | *1.00* | *1.00* |
| Former smoker |  | -0.054 | -0.053 |
|  |  | (-0.121, 0.014) | (-0.121, 0.014) |
| Current smoker |  | -0.127* | -0.123* |
|  |  | (-0.224, -0.029) | (-0.221, -0.026) |
| Never drinks alcohol *(omitted)* |  | *1.00* | *1.00* |
| 1-7 drinks per week |  | -0.040 | -0.039 |
|  |  | (-0.108, 0.029) | (-0.106, 0.029) |
| 8-14 drinks per week |  | -0.069 | -0.066 |
|  |  | (-0.172, 0.034) | (-0.168, 0.037) |
| 15 or more drinks per week |  | -0.149 | -0.144 |
|  |  | (-0.309, 0.010) | (-0.304, 0.015) |
| History of a head injury |  | -0.056 | -0.058 |
|  |  | (-0.224, 0.111) | (-0.226, 0.111) |
| Self-assessed hearing^c^ |  | -0.011 | -0.011 |
|  |  | (-0.046, 0.023) | (-0.045, 0.024) |
| Self-assessed vision^c^ |  | -0.034* | -0.034* |
|  |  | (-0.068, -0.000) | (-0.068, -0.001) |
| Hypertension |  | 0.008 | 0.008 |
|  |  | (-0.066, 0.082) | (-0.066, 0.082) |
| Medication for high cholesterol |  | 0.005 | 0.007 |
|  |  | (-0.081, 0.092) | (-0.079, 0.094) |
| Underweight (BMI < 18.5) |  | -0.351 | -0.348 |
|  |  | (-0.706, 0.005) | (-0.704, 0.008) |
| Normal (BMI 18.5-24.9) *(omitted)* |  | *1.00* | *1.00* |
| Overweight (BMI 25-29.9) |  | 0.015 | 0.012 |
|  |  | (-0.059, 0.089) | (-0.062, 0.086) |
| Obese, Class I (BMI 30-34.9) |  | 0.003 | 0.001 |
|  |  | (-0.091, 0.096) | (-0.092, 0.094) |
| Obese, Class II/III (BMI 35+) |  | 0.078 | 0.076 |
|  |  | (-0.040, 0.195) | (-0.042, 0.193) |
| Heart problems |  | 0.024 | 0.024 |
|  |  | (-0.062, 0.109) | (-0.061, 0.110) |
| Stroke |  | -0.037 | -0.032 |
|  |  | (-0.252, 0.177) | (-0.246, 0.181) |
| Diabetes |  | -0.078 | -0.077 |
|  |  | (-0.206, 0.050) | (-0.205, 0.051) |
| Physical limitations^c^ |  | -0.021 | -0.020 |
|  |  | (-0.060, 0.017) | (-0.059, 0.018) |
| Depressive symptoms^c^ |  | 0.002 | 0.002 |
|  |  | (-0.031, 0.036) | (-0.032, 0.035) |
| Extraversion^c^ |  | 0.016 | 0.017 |
|  |  | (-0.024, 0.056) | (-0.022, 0.057) |
| Conscientiousness^c^ |  | 0.004 | 0.004 |
|  |  | (-0.029, 0.037) | (-0.029, 0.036) |
| Neuroticism^c^ |  | -0.011 | -0.009 |
|  |  | (-0.045, 0.024) | (-0.043, 0.026) |
| Openness^c^ |  | -0.001 | -0.002 |
|  |  | (-0.041, 0.038) | (-0.041, 0.036) |
| Agreeableness^c^ |  | -0.026 | -0.024 |
|  |  | (-0.062, 0.011) | (-0.061, 0.012) |
| Constant | -0.553*** | -0.456*** | -0.453*** |
|  | (-0.594, -0.511) | (-0.581, -0.331) | (-0.577, -0.330) |

Abbreviations: BMI = Body Mass Index.

^a^ There was wide variation in the length of the interval between Waves 2 and 3 cognitive testing (mean=9.6 years, range=7.5 to 13.6). Consequently, we adjusted the observed change in cognition to represent the implied change after 10 years of aging (e.g., divided by the length of the interval between the two cognitive tests and multiplied by 10).

^b^ Age was measured in decades after age 65: (age – 65/10); thus, the coefficient represents the effect per 10 years of aging.

^c^ Frequency per month.

^d^ Standardized so that the coefficient represents the effect per SD.

Supplementary Table 6. Estimated levels of cognition and cognitive decline between selected ages, MIDUS

|  | **Cognition Age 35:** | **Cognitive Decline across 10-Year Age Intervals:** | | | | | **Cognition at Age 85:** | |
| --- | --- | --- | --- | --- | --- | --- | --- | --- |
|  |  | **35 to 45** | **45 to 55** | **55 to 65** | **65 to 75** | **75 to 85** |  |  |
| **Based on Model 2:** |  |  |  |  |  |  |  | |
| Mean frequencies of activities^a^ *[reference group]* | 0.26 | -0.08 | -0.16 | -0.28 | -0.46 | -0.68 | -1.32 | |
| Frequency by activity type |  |  |  |  |  |  |  |  |
| Cognitive activities: 1 SD above mean^b^ | **0.43***** | -0.09 | -0.17 | -0.30 | -0.47 | -0.69 | **-1.15***** | |
| Physical activity: 1 SD above mean^b^ | **0.30*** | -0.08 | -0.16 | -0.28 | -0.46 | -0.68 | **-1.28*** | |
| Social contacts: 1 SD above mean^b^ | 0.26 | -0.06 | -0.14 | -0.27 | -0.44 | -0.66 | -1.32 | |
| Social group participation: 1 SD above mean^b^ | 0.27 | -0.10 | -0.17 | -0.30 | -0.47 | -0.69 | -1.31 | |
| **Based on Model 3:** |  |  |  |  |  |  |  | |
| Mean activity diversity^a^ *[reference group]* | 0.13 | -0.07 | -0.15 | -0.28 | -0.45 | -0.68 | -1.42 | |
| Activity diversity: 1 SD above mean^b^ | **0.19**** | -0.07 | -0.15 | -0.28 | -0.45 | -0.67 | **-1.36**** | |

*Note*. Estimated values of cognition are expressed in SD units. The estimates for cognition at ages 35 and 85 are based on Table 2, whereas the estimates for 10-year cognitive decline are based on Supplementary Table 5. Values that were significantly better (higher level of cognition or slower cognitive decline) than the reference group are shown in bold.

^a^ Represents the reference group with average levels of all continuous variables (including the frequency of all activity domains or diversity) and the omitted category for binary and categorical variables (i.e., Non-Hispanic White men who were married, completed high school or GED, currently employed, never smoked, never drink alcohol, do not have hypertension, do not take medication for high cholesterol, and did not have a history of heart problems, a history of a stroke, or diabetes).

^b^ Same as above except for the specified difference in the indicated activity measure.

* *p* < 0.05; ** *p* < 0.01; *** *p* < 0.001 relative to the reference group

Supplementary Figure 1. Directed acyclic graph (DAG) for the prospective relationship between activity engagement and subsequent cognitive decline

**Observed Potential**

**Confounders**

Physical

Activity

Cognitive

Activities

Subsequent

Aging-Related

Cognitive Decline

Social Group

Participation

Social Contact

with Family

& Friends

**Unobserved**

**Confounders**

Note: This study aimed to estimate the total causal effect of activity engagement (measured at baseline) on subsequent cognitive decline. We included four types of activities: cognitive, physical, social contact with family and friends, and participation in social groups (e.g., clubs, community organizations, religious attendance). The observed potential confounders comprised demographic characteristics (age, sex, race/ethnicity) and baseline measures of social‑economic context (educational attainment, marital status, employment status), personality traits, other health behaviors (smoking, alcohol consumption), and health status (history of a head injury, hearing, vision, diabetes, hypertension, high cholesterol, obesity, history of heart problems, history of stroke, physical limitations, and depression); see Supplementary Methods Section 6 for more details regarding the confounders. The unobserved confounders could include early life cognition, aging‑related cognitive decline prior to baseline, activity levels earlier in life, and lifetime exposure to air pollution; dashed lines denote unobserved pathways.

Supplementary Figure 2. Estimated age trajectory of cognition by selected levels of physical activity, HRS


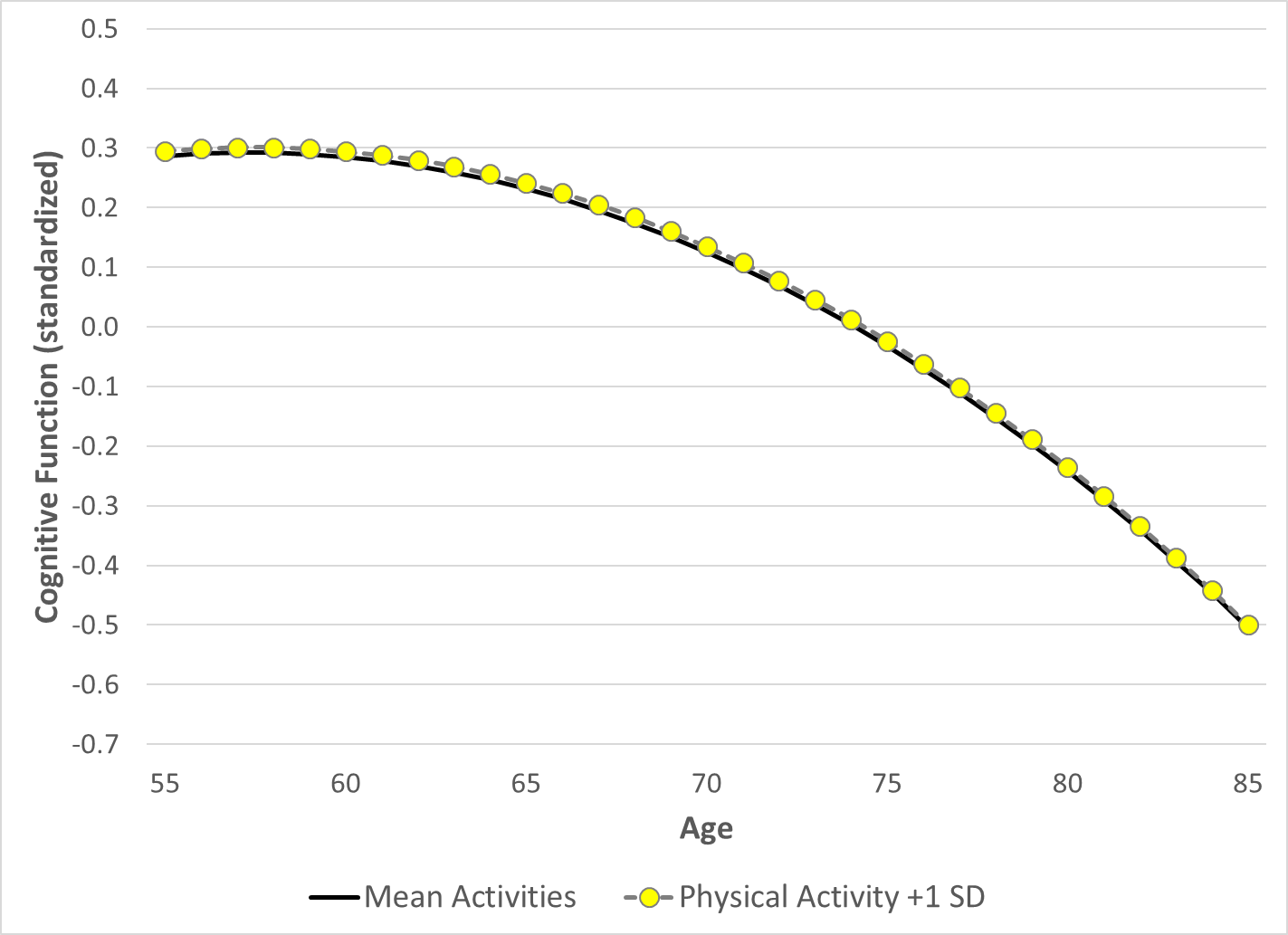


*Note*. The y-axis represents cognition (standardized to represent SD units). The estimates are based on the coefficients from Model 3 (Table 1). The black line represents the reference group (i.e., mean levels of all continuous variables including activity frequencies and diversity and the omitted category for binary and categorical variables). For the line marked by yellow circles, all values remain unchanged except for the specified difference in physical activity.

Supplementary Figure 3. Estimated age trajectory of cognition by selected levels of social contact and social group participation, HRS


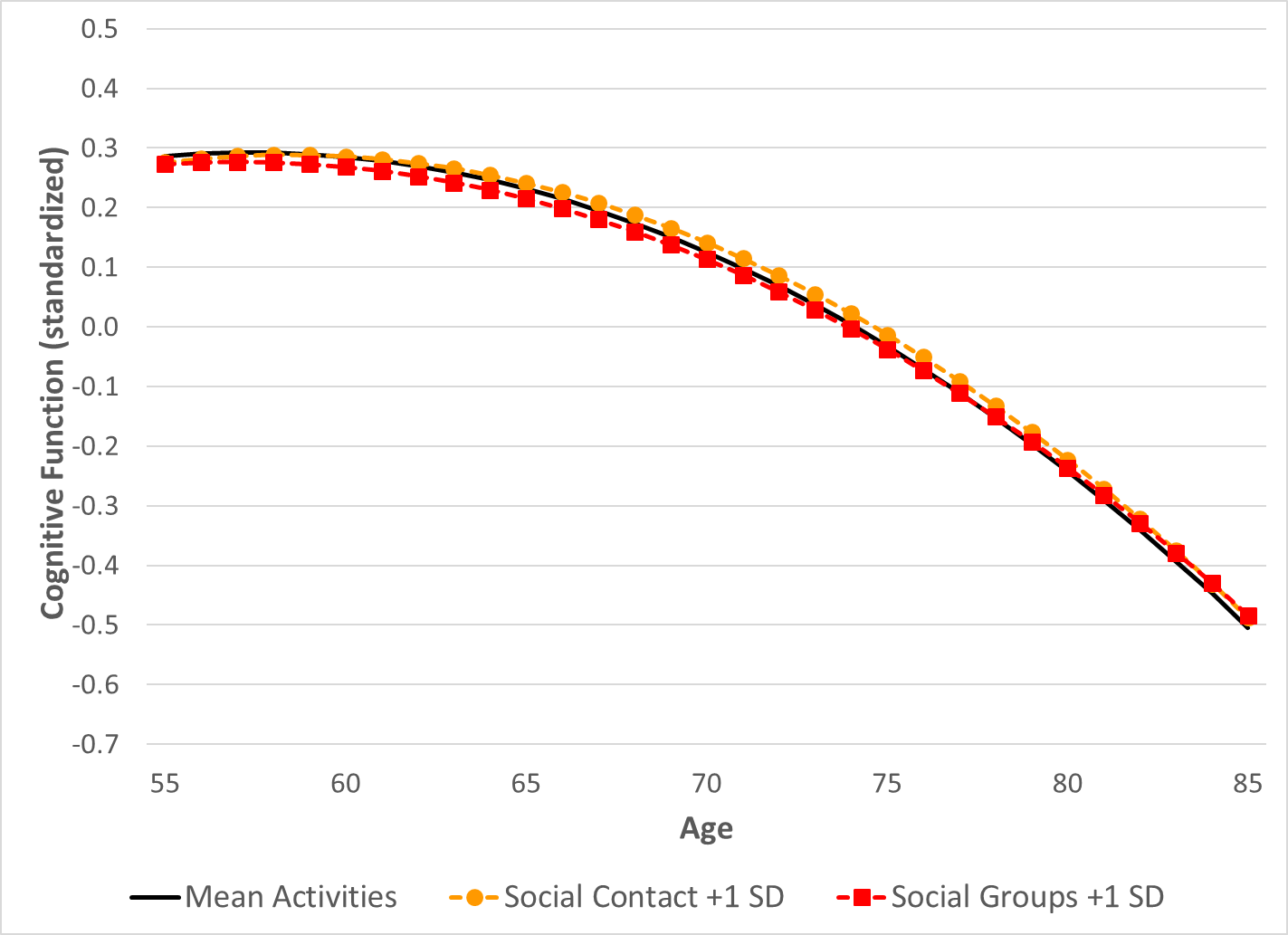


*Note*. The y-axis represents cognition (standardized to represent SD units). The estimates are based on the coefficients from Model 2 (Table 1). The black line represents the reference group (i.e., mean levels of all continuous variables including activity frequencies and diversity and the omitted category for binary and categorical variables). For the other trajectories, all values remain unchanged except for the specified difference in the indicated activity measure.

1. Although MIDUS targeted Americans aged 25-74, the final sample included a few respondents aged 20-24 (*N*=18) or 75 (*N*=5) at the time of the Wave 1 phone interview. [↑](#footnote-ref-1)
2. We did not include air pollution because these surveys did not collect information the history of exposure to air pollution. [↑](#footnote-ref-2)
